# High-affinity and selective detection of pyrophosphate in water by a resorcinarene salt receptor†
†Electronic supplementary information (ESI) available: Full analytical methods, experimental details, NMR, ITC and UV-vis/fluorescence data and detailed conformational search and the applied computational methods. See DOI: 10.1039/c7sc05167k


**DOI:** 10.1039/c7sc05167k

**Published:** 2017-12-19

**Authors:** Ngong Kodiah Beyeh, Isabel Díez, S. Maryamdokht Taimoory, Daniel Meister, Andrew I. Feig, John F. Trant, Robin H. A. Ras, Kari Rissanen

**Affiliations:** a Aalto University , School of Science , Department of Applied Physics , Puumiehenkuja 2 , FI-02150 , Espoo , Finland . Email: tbeyeh@gmail.com ; Email: robin.ras@aalto.fi; b University of Windsor , Department of Chemistry and Biochemistry , Windsor , ON N9B 3P4 , Canada . Email: j.trant@uwindsor.ca; c Aalto University , School of Chemical Engineering , Department of Bioproducts and Biosystems , Kemistintie 1 , 02150 Espoo , Finland; d Wayne State University , Department of Chemistry , 5101 Cass Ave. , Detroit , MI 48202 , USA; e University of Jyvaskyla , Department of Chemistry , P. O. Box 35 , FI-40014 Jyväskylä , Finland . Email: kari.t.rissanen@jyu.fi

## Abstract

Pyrophosphate (PPi) is a byproduct of DNA and RNA synthesis, and abnormal levels are indicative of disease. We report the high-affinity binding of PPi in water by *N*-alkyl ammonium resorcinarene chloride receptors. Experimental analysis using ^1^H and ^31^P NMR, isothermal titration calorimetry, mass spectrometry, and UV-vis spectroscopy all support exceptional selectivity of these systems for PPi in water. The measured affinity of *K*_1_ = 1.60 × 10^7^ M^–1^ for PPi is three orders of magnitude larger than that observed for binding to another phosphate, ATP. This exceptional anion-binding affinity in water is explored through a detailed density functional theory computational study. These systems provide a promising avenue for the development of future innovative medical diagnostic tools.

## Abstract 2

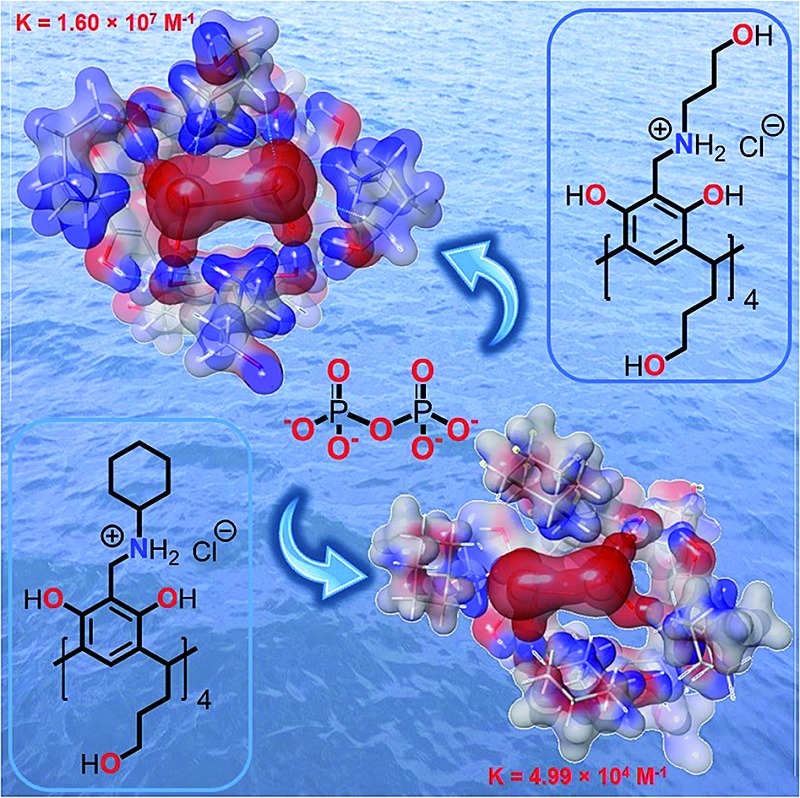
 *N*-Alkyl ammonium resorcinarenes selectively bind pyrophosphate in pure water with an exceptionally high binding constant of up to 1.60 × 10^7^ M^–1^, three orders of magnitude higher than ATP.

## Introduction

Considerable effort is continuously being invested in developing receptors to detect biologically relevant ions under physiological conditions.^1^ Such receptors, utilizing an array of weak interactions, could be used in the design of functional assemblies with far-reaching applications in biomedicine including as sensors, transport agents and as drugs.^2^–^5^ Non-covalent interactions form the basis of molecular recognition between hosts and guests, and are especially relevant for ion-paired systems. Biologically-relevant anions are of significant interest:^6^,^7^ many cofactors, most enzyme substrates and DNA are all anionic in nature.^8^ Anions such as pyrophosphate (PPi) and adenosine triphosphate (ATP) are key intermediates for energy transduction and are common to a number of essential metabolic processes.^6^,^7^ When the ratio of these anions falls out of balance, manifested as abnormal levels of PPi, abnormal physiology can result. A number of diseases are strongly associated with elevated PPi levels, including cancer, arthritis, crystal deposition disease, and Paget's disease.^9^ Significant recent research effort has been focused on developing more potent PPi sensors for early diagnosis of these conditions;^10^–^18^ this includes a terpyridine–Zn^2+^ complex capable of nM PPi detection previously reported by our group.^10^ However, most of these chemosensors require a metal ion to mediate sufficient sensitivity.^10^–^13^ Very high binding constants have been obtained using metal-free chemosensors, but only in organic media.^18^ For example, Schanze and coworkers have developed a conjugated polyamine fluorescent probe capable of sensing PPi at concentrations as low as 100 μM.^19^ However, any simple clinical application would require detection from serum in an aqueous environment. The high hydration energies of anions in water requires that any receptor–anion interaction be very strong to overcome the solvation energy of the anion as the very strong, charge-assisted anion–water hydrogen bonds need to be broken.^20^,^21^ The careful design and control of non-covalent interactions strong enough to accomplish this objective is an enormous challenge to researchers at both fundamental and application levels.^22^ This is especially true for doubly and/or densely charged anions such as PPi. Multiple hydrogen-bonding (H-bonding) interactions to a well-defined binding site in a protein cavity, whereby the directed H-bonds complement the shape of the anion, are usually responsible for anion binding in natural systems.^21^–^25^ In nature, the key binding pocket is usually located in a relatively hydrophobic environment that facilitates anion affinity, as the water is slowly removed from the solute as it enters the binding cavity. Synthetic organic receptors however do not often work on the same size scale and are condemned to rely mostly on H-bonding interactions for anion binding; this normally makes them uncompetitive in water, and studies are often restricted to aprotic media where the anion desolvation energies are much lower.^26^ In combination with H-bonding, strong electrostatic or metal–ligand interactions have therefore been required to overcome the anion hydration energy and allow anion binding in water and/or biological fluids.

*N*-Alkyl ammonium resorcinarene halides (NARXs),^27^,^28^ are large organic salt receptors where the four spatially fixed halides anions are held in place by the strong circular intramolecular H-bond seam (···NArRH2^+^···X^–^···)_4_ creating a cavity with a size and shape analogous to traditional covalent resorcinarene cavitands.^29^,^30^ While possessing deeper cavities when compared to regular resorcinarenes, the circular cation–anion seam introduces a second local binding site as observed in the reported crystal structures.^27^–^32^ This is especially true when the counter anions are either chlorides or bromides.^33^ Moreover, different upper rim functional groups can significantly influence the binding abilities of the cavitand as a whole.^31^,^32^ The NARXs have been used as supramolecular receptors for neutral^31^,^32^ and anionic^33^,^34^ guests in organic media and as synthons for larger supramolecular architectures held together by halogen bonds.^35^ The insolubility of the NARXs in water has limited their application to biological processes. However, decorating the NARXs with four terminal hydroxyl groups on either the upper or lower rim makes them water soluble while maintaining the hydrophobic cavity and the hydrophilic cation–anion seam. Recent results show these water soluble NARXs effectively bind hydrocarbons, halocarbons and viologen derivatives in water.^36^

**Fig. 1:**
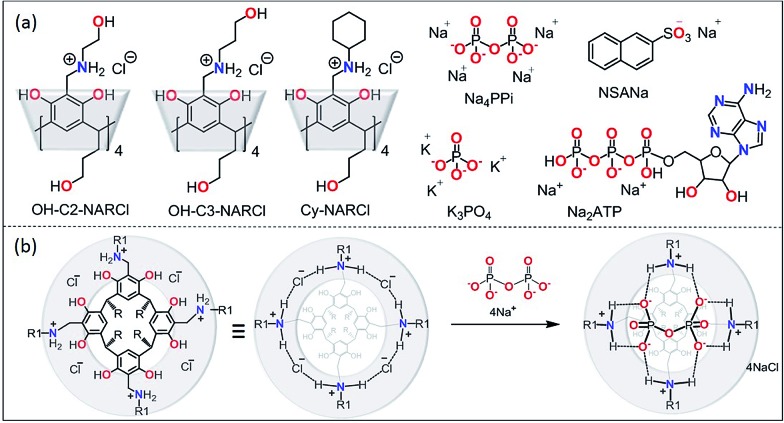
(a) The receptors OH-C2-NARCl, OH-C3-NARCl and Cy-NARCl, the phosphates K_3_PO_4_, Na_4_PPi and Na_2_ATP, and the guest NSANa; (b) schematic showing the interaction between PPi and NARCl. Note on nomenclature: for clarity, the number of residual chlorides after complexation are omitted in the structure names in the text. For PPi (PPi@NAR), there are no residual chlorides; however, for ATP (ATP@NAR) the more accurate form is ATP@NARCl_2_, while for PO_4_^3–^ (PO_4_^3–^@NAR) the more accurate form is (PO_4_^3–^@NARCl_1_).

There is a huge potential for anion receptors that can incorporate both electrostatic and H-bond interactions, and can operate in water.^23^ The phosphate anions are of particular interest due to their importance in biological processes; however, it can be difficult to differentiate between the different species in solution (phosphate, PPi, ATP, ADP *etc.*). Though there are many polycationic receptors that can bind phosphate anions such as polycationic crown ethers,^37^ and polycationic TREN receptors,^38^ the nature of the binding is not specific for the shape of PPi. The NARXs, however, are tetracationic in nature and their diameter and circular shape complements the PPi tetra-anion along the circular intramolecular H-bond seam (···NArRH2^+^···X^–^···)_4_ of the NARXs. We hypothesize that the NARXs will be suitable receptors for PPi in water since they possess complementary binding characteristics such as size fit, electrostatic and H-bond properties as well as several other attractive interactions. In this contribution, we report the selective and high-affinity binding of PPi by three water-soluble NARCls (Fig. 1); one with a more flexible terminal hydroxyl propyl group at the upper rim (OH-C3-NARCl), the second with a shorter and less flexible terminal hydroxyl ethyl group at the upper rim (OH-C2-NARCl), and the third with a rigid cyclohexyl group at the upper rim (Cy-NARCl). In addition to PPi, the binding properties of the receptors towards a tribasic monophosphate (K_3_PO_4_), and a dibasic triphosphate (ATP) are also investigated. Quantification of the binding was carried out through computational studies and a series of Isothermal Titration Calorimetry (ITC) and ^1^H NMR experiments with the results revealing a particularly high binding constant (*K*_1_ = 1.60 ± 0.77 × 10^7^ M^–1^) between the OH-C3-NARCl receptor and PPi. The PPi affinity was further probed using UV-vis and additional NMR studies through guest displacement experiments with 2-naphthalenesulfonic acid sodium salt (NSANa).^39^,^40^ These studies were corroborated in the gas phase *via* electrospray ionization mass spectrometry (ESI-MS), and the binding modes were justified using density functional theory (DFT) calculations at the (B3LYP/6-31G**) level of theory.

## Results and discussion

In a recent study in organic media,^33^ the NARX receptors were shown to be highly selective for the chloride anion over other anions such as bromide, iodide, nitrate, triflate and picrate. The specificity towards chloride was determined to be due to both its suitable size, it fits perfectly between two adjacent ammonium moieties, as well as its strong H-bond acceptor behaviour. The NARXs are tetra-cationic and the halide counteranions adopt preferred positions around the circular hydrogen-bonded cation–anion seam. These receptors can bind neutral compounds through CH···π interactions with the lower cavity and H-bonds with the cation–anion seam. A perfectly sized anion could interact with these receptors at the cation–anion seam. By inspection, PPi appeared to be the perfect guest (Fig. 1b).

### NMR spectroscopy

**Fig. 2:**
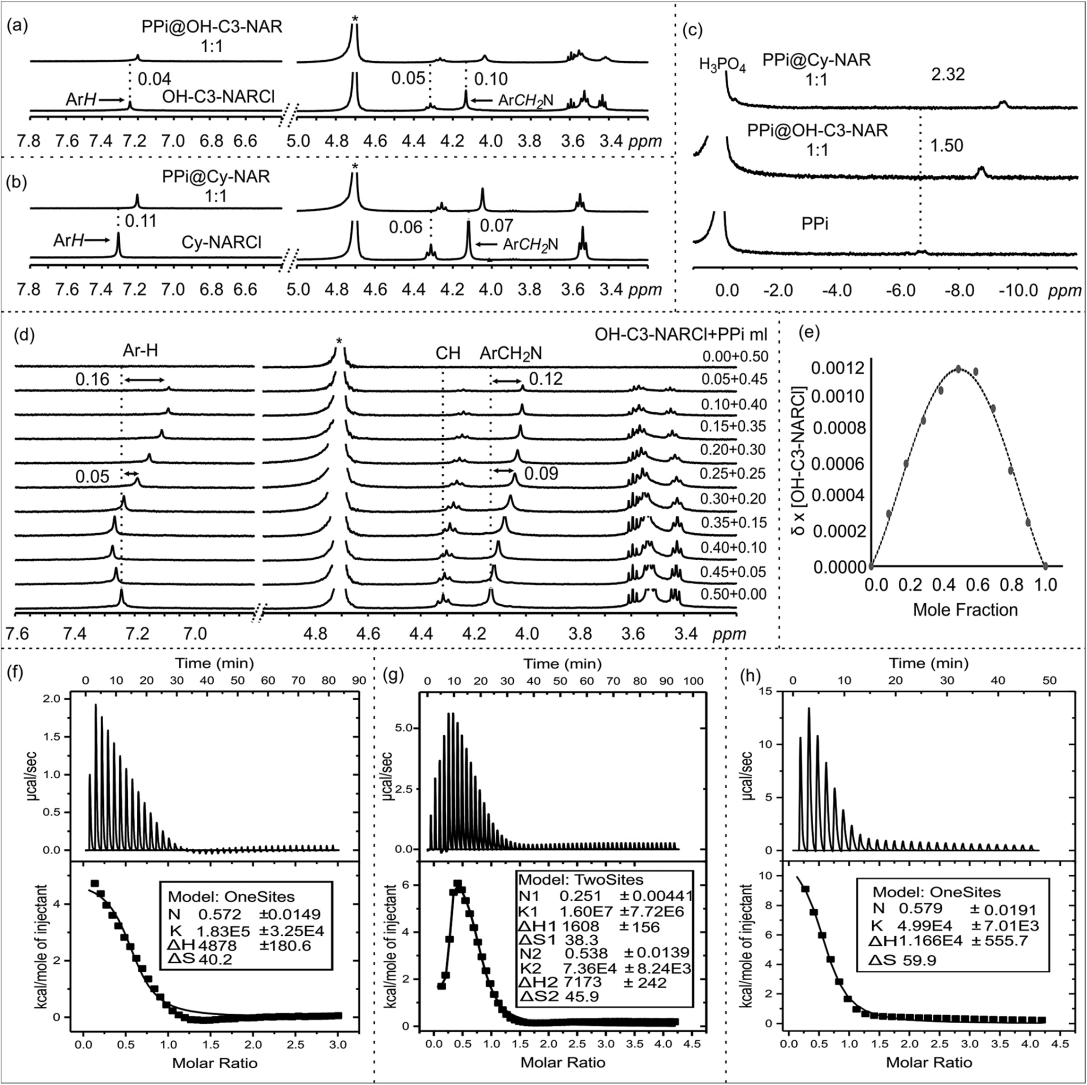
NMR spectra in D_2_O at 298 K: ^1^H NMR of (a) OH-NARCl and the equimolar mixture OH-C3-NARCl and PPi, (b) Cy-NARCl and the equimolar mixture Cy-NARCl + PPi. (c) ^31^P NMR spectra of PPi, and equimolar mixtures OH-C3-NARCl + PPi, and Cy-NARCl + PPi, with H_3_PO_4_ as the external standard. (d) ^1^H NMR spectra of different equivalents of OH-C3-NARCl and PPi for Job plot calculation. The dashed lines give an indication of the signal changes in ppm. Star represents the residual D_2_O solvent. (e) Job plot of OH-C3-NARCl and PPi revealing a mainly 1 : 1 binding stoichiometry. ITC traces of the titration of the receptors (5 mM) into PPi (0.25 mM) in H_2_O at 303 K: (f) OH-C2-NARCl, (g) OH-C3-NARCl and (h) Cy-NARCl.

The anion binding behaviour of the NARCls towards three different phosphate anions (PO_4_^3–^, PPi and ATP) was analyzed in solution through multiple NMR experiments. ^1^H NMR spectra of the pure guests and the pure NARCl receptors in D_2_O were recorded at 298 K. ^1^H NMR spectra of equimolar mixtures of the NARCl receptors and guests in D_2_O were also recorded at 298 K. The binding processes are fast on the NMR timescale. Clear changes in the hosts' signals confirm host–guest interaction between the NARCls and the phosphates (Fig. 2, S2 and S3†). Due to H/D exchange in D_2_O, the OH and NH_2_ signals associated with the groups expected to bind the phosphates could not be monitored.

However, up to 0.11 ppm upfield shifts are observed for the aromatic protons, and up to 0.10 ppm upfield shifts can be noted for the methylene protons closest to the ammonium groups of the receptors. These changes in the host's signals clearly support the host re-organizing the cavity during the binding process. Multiple ^31^P NMR experiments were carried out to probe possible changes in the phosphorus signals. Significant upfield shifts of the ^31^P signals of PO_4_^3–^ and PPi confirmed encapsulation of the phosphate anions (Fig. 2c and S4–S6†). Strangely, only negligible changes in the ^31^P signals were observed with the ATP anion (Fig. S6†).

Determining the binding stoichiometry of a receptor-substrate ensemble is very important to understand the binding process. The method of continuous variation (Job's method) is a standard technique for this measurement.^41^ However, the approach is only recognized as being diagnostic for 1 : 1 binding modes; it is not considered a reliable technique for precisely quantifying higher-order binding modes.^41^ Determination of the binding stoichiometry through a series of Job plot experiments revealed a 1 : 1 complex for all the phosphate anions (Fig. 2d and e, S7–S9†).

### Isothermal titration calorimetry (ITC)

The binding interactions between the NARCl receptors and the three phosphate anions were quantified through a series of ITC experiments in H_2_O (Fig. 2f, S10 and S11†). The ITC data from the interaction between the NARCls and PO_4_^3–^ could not be fitted to any model. This result is best interpreted as nonspecific binding of the monophosphate. Such an interpretation is logical taking into account the smaller size of PO_4_^3–^ as compared to PPi and ATP.

The thermodynamic parameters of host–guest binding (*K*, Δ*H*, Δ*S*, and Δ*G*) between the NARCls and the other phosphate anions (PPi and ATP) were determined from fitting the ITC data to a single binding site model (Table 1). Surprisingly, the binding data derived from OH-C3-NARCl and PPi could only be fitted to a two-site binding model (Fig. 2g). A detailed analysis of the binding isotherm reveals there are clearly two binding processes occurring.^42^ Negative Δ*G* values reveal the binding of both PPi and ATP to be spontaneous at 303 K. The Δ*H* and *T*Δ*S* results indicate that complexation of PPi is entropy driven while for ATP, it is largely entropy driven with small enthalpic contributions. PPi is assumed to be heavily hydrated in water.^43^ Upon complexation with the receptors, the solvation sphere around the PPi is disrupted making the binding process endothermic.^43^ Also, binding process with an ion exchange process, releases the counterions (Na^+^ and Cl^–^) from the dissolved complex which is the other factor for the observed process being largely entropy driven. This has been previously observed for other supramolecular binding interactions between poly(ammonium chlorides) and poly(sodium phosphates), and may be due to the particularly strong ion pairs in the parent NARCl host.^44^

**Table 1:** Thermodynamic binding parameters of formed complexes between the NARCl receptors and the phosphate guests by ITC^*a*^

Complex	*K*_1_ (×10^4^) M^–1^	Δ*H*_1_ kcal mol^–1^	*T*Δ*S*_1_ kcal mol^–1^	Δ*G*_1_ kcal mol^–1^
PPi@OH-C3-NARCl	1600 ± 770	1.61 ± 0.15	11.60	–10.00
PPi@OH-C2-NARCl	18.30 ± 3.25	4.82 ± 0.18	12.18	–7.30
PPi@Cy-NARCl	4.99 ± 0.70	11.66 ± 0.55	18.15	–6.49
ATP@OH-C3-NARCl	1.22 ± 0.36	–0.41± 0.07	5.24	–5.65
ATP@Cy-NARCl	0.49 ± 0.11	–0.51± 0.07	4.61	–5.12

Complex	*K*_2_ (×10^4^) M^–1^	Δ*H*_2_ kcal mol^–1^	*T*Δ*S*_2_ kcal mol^–1^	Δ*G*_2_ kcal mol^–1^
PPi@OH-C3-NARCl	7.36 ± 0.82	7.17 ± 0.24	13.91	–6.73

^*a*^ITC was done in H_2_O at 303 K.

The *K*_1_ values reveal generally higher binding constant for PPi over ATP. The interaction between OH-C3-NARCl and PPi reveals a particularly high binding constant for the first of two binding processes (*K*_1_ = 1.60 ± 0.77 × 10^7^ M^–1^ and *K*_2_ = 7.36 ± 0.82 × 10^4^ M^–1^) and the data well approximates a two-site binding model. As the cavity is committed to the binding of the first PPi molecule, this second interaction is most likely an allosteric binding to the terminal hydroxyls either above or below the rim. To find out which rim is responsible for binding, we used Cy-NARCl having hydroxyl groups only on the lower rim. In case of Cy-NARCl no such tertiary interaction was observed. Interestingly, no tertiary interaction was observed with the OH-C2-NARCl receptor, which is only one carbon shorter on the upper rim substituents, either. Consequently, it is very likely that for OH-C3-NARCl, binding occurs with the more flexible upper hydroxyl chains. As these hydroxyl groups are spatially close to the cavity, it is unsurprising that the two PPi molecules would have an electrostatic repulsive effect, which could explain part of the observed rise in system energy. It is particularly interesting that the binding of PPi by the OH-C3-NARCl (*K*_1_ = 1.60 ± 0.77 × 10^7^ M^–1^) is two orders of magnitude higher than with the shorter arm OH-C2-NARCl (*K*_1_ = 1.83 ± 0.32 × 10^5^ M^–1^) and three orders of magnitude higher than the more rigid Cy-NARCl (*K*_1_ = 4.99 ± 0.70 × 10^4^ M^–1^). The upper rim terminal hydroxyl groups in combination with the flexible propyl spacer wraps around the PPi anion with additional hydrogens thus resulting in a tightly bound assembly, and supports the results from the computational calculations that will be discussed later. The lower binding values (10^4^ M^–1^ and 10^3^ M^–1^) for ATP can be attributed to a less than complementary size fit with the larger anion.

As ITC is a thermodynamic measurement, and as both the receptors and guests are salts leading to solutions with varying pHs (see Table S1†), we repeated the binding assay in Tris buffer, pH 7.10. Despite this competitive buffering salt being in large excess, we still observed a significant binding interactions (Fig. S19:† 10 mM Tris, PPi@OH-C3-NAR, *K* = 3.54 ± 0.38 × 10^4^ M^–1^, PPi@Cy-NAR, *K* = 3.15 ± 0.47 × 10^4^ M^–1^; 50 mM Tris, PPi@OH-C3-NAR, *K* = 2.75 ± 0.21 × 10^2^ M^–1^, PPi@Cy-NAR, *K* = 1.14 ± 0.50 × 10^2^ M^–1^) indicating that the observed isotherms do not arise from simple protonation–deprotonation events, and thus suggests that we are observing true host–guest interactions.

### Mass spectrometry

Electrospray ionization mass spectrometry (ESI-MS) is a soft ionization technique extensively used for structure determinations and analyses of complex species in the gas phase.^45^ ESI-MS often has trouble detecting intermolecular host–guest interactions due to the high energy of the process; unless the binding is exceptionally strong, it is rare to observe the complexes as they can easily dissociate in high energy systems. In case of OH-C3-NARCl/PPi in the negative ion mode, the complex was easily detected, providing further evidence for the strength of this interaction. The NARXs are held together by several weak interactions, emphasized by the many species usually seen in their traditional mass spectra.^31^–^33^

**Fig. 3:**
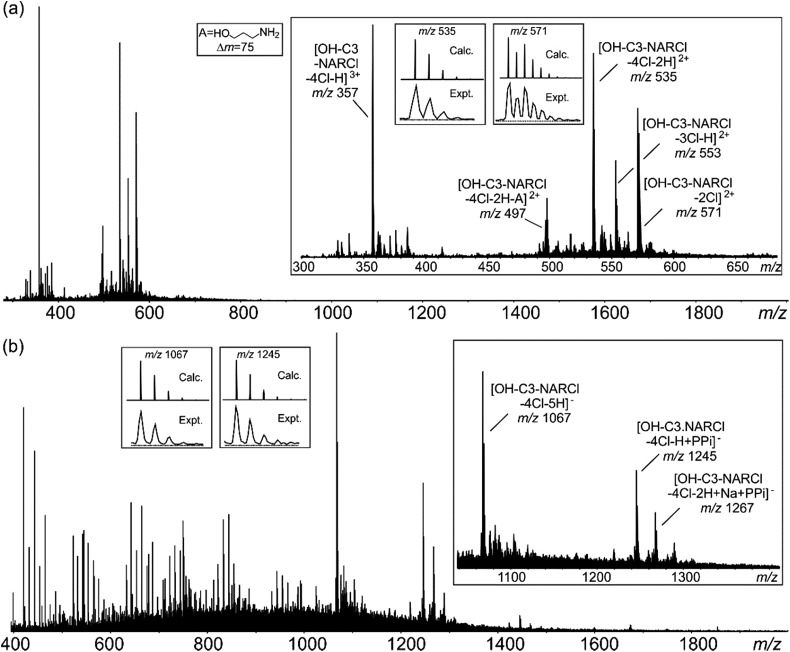
(a) Positive ESI mass spectrum of OH-C3-NARCl (20 μM). (b) Negative ESI mass spectrum of an equimolar mixture (20 μM) of OH-C3-NARCl and PPi. Insets: experimental and calculated isotope patterns of selected signals.

The samples (2 mM) were prepared in H_2_O and then diluted into methanol (20 μM). In the spectrum of the pure OH-C3-NARCl receptor in the positive ion mode, progressive losses of HCl resulted in mainly doubly charged species such as at *m*/*z* 571, 553 and 535 (Fig. 3a, Table 2). Intramolecular H-bonding between one OH group on the resorcinol ring and the ammonium nitrogen supports a 1,4-elimination of an amine which proceeds through a six-membered transition structure (Fig. 3a, Scheme S3, Fig. S15†).^46^ This 1,4-elimination of amine (A) is observed in the mass spectrum of OH-C3-NARCl at *m*/*z* 497 (Fig. 3a, Table 2). With all four chlorides of the OH-C3-NARCl receptor being replaced by the PPi tetra-anion, it is challenging to ionize this charge neutral complex in the positive ion mode. A weak signal corresponding to the sodium adduct is observed at *m*/*z* = 1269 (Fig. S13,† Table 2). However, the hydroxyl groups are easily deprotonated in the negative ion mode. Clear signals corresponding to the 1 : 1 complex at *m*/*z* 1245 and 1267 are observed (Fig. 3b, Table 2). With the phosphate anion, the loss of the four chlorides with the trianionic PO_4_^3–^ resulted in a 1 : 1 complex in the positive ion mode and is observed at *m*/*z* 1167, and 1205 (Fig. S14,† Table 2). ATP has several nitrogen groups that could easily be ionized. Signals corresponding to the 1 : 1 complex with ATP are observed in the positive ion mode, for example at *m*/*z* 1576 and 799 (Fig. S15,† Table 2). The isotope patterns obtained by experiment agree with those simulated on the basis of natural abundances.

**Table 2:** ESI-MS ions of the different species observed in the gas phase

Ion	*m*/*z*	Ion	*m*/*z*
[OH-C3-NARCl-2Cl]^2+^	571	[OH-C3-NARCl-4Cl + Na + PPi]^+^	1269
[OH-C3-NARCl-3Cl-H]^2+^	553	[OH-C3-NARCl-4Cl-H + PPi]^–^	1245
[OH-C3-NARCl-4Cl-2H]^2+^	535	[OH-C3-NARCl-4Cl-2H + Na + PPi]^–^	1267
[OH-C3-NARCl-4Cl-2H-A]^2+^	497	[OH-C3-NARCl-4Cl + PO_4_]^+^	1167
[OH-C3-NARCl-4Cl + H + ATP]^+^	1576	[OH-C3-NARCl-4Cl-H + Na + PO_4_]^+^	1205
[OH-C3-NARCl-4Cl + H + Na + ATP]^2+^	799		

### Guest displacement assay

ITC results clearly show the PPi to be the most strongly bound guest with a binding constant two orders of magnitude higher in the case with OH-C3-NARCl than with the other host–guest complexes. ^1^H NMR competition experiments were done to investigate guest preference between PPi and ATP. In the experiments, to an equimolar mixture of OH-C3-NARCl and PPi, or OH-C3-NARCl and ATP, one equivalent of the other guest was added and the ^1^H NMR recorded. The results were then compared to when no competing guest is present. In the equimolar mixture between OH-C3-NARCl and ATP, following the methylene protons ArCH_2_N closest to the binding site, clear changes show PPi to replace ATP (Fig. S12†). The reverse process shows ATP could not replace PPi once the PPi-NARX complex is established (Fig. S12†).

**Fig. 4:**
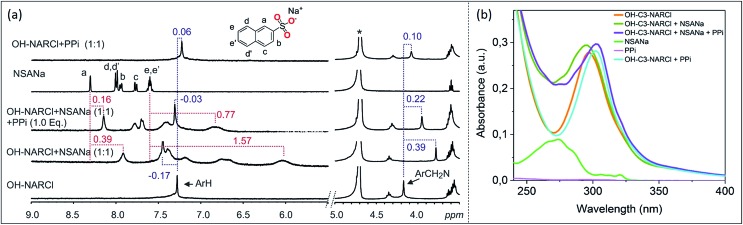
(a) ^1^H NMR spectra (D_2_O, 298 K) of competition experiment between OH-C3-NARCl towards ATP and PPi. Results show PPi is the preferred guest. The dashed lines give an indication of the signal changes in ppm. Star represent the residual D_2_O solvent. (b) Absorption spectra showing the spectral changes in the receptor after the addition of first NSANa (blue-shift) and then PPi (red-shift) as guests. Spectra were recorded by transmission UV-vis spectroscopy in a 1 cm cuvette with a receptor concentration of 20 μM where 1 equivalent of the guest was added under stirring.

Detection and differentiation of analytes by supramolecular receptors is very challenging. Indicator displacement assays (IDAs) with synthetic receptors offer a supramolecular approach for the detection of bioanalytes.^39^,^40^ The detection of PPi by OH-C3-NARCl in water was further analysed through an IDA with 2-naphthalenesulfonic acid sodium salt (NSANa). We anticipate that the interaction between OH-C3-NARCl and PPi is stronger than the interaction between OH-C3-NARCl and NSANa. As such, NSANa could be used as an indicator to show the preference for, and detection of, PPi. In the experiments, an equimolar mixture (2.5 mM) of OH-C3-NARCl and NSANa was prepared and the ^1^H NMR measured. High upfield shifts of the NSANa signals (up to 1.57 ppm) clearly show the NSANa to be located deep into the cavity of the receptor (Fig. 4a). Also, the aromatic protons of the receptor are de-shielded (–0.17 ppm) which is in line with the receptor modulating its internal cavity to accommodate the larger NSANa guest. To this equimolar OH-C3-NARCl and NSANa mixture, one equivalent of PPi was then added. All the shielded signals of the NSANa guest start de-shielding towards the free species clearly suggesting a huge preference for PPi. Also, the de-shielded aromatic signals of the receptors are then shielded towards the PPi@OH-C3-NAR species (Fig. 4a).

### Computational study (conformational search and noncovalent interaction (NCI) analysis)

**Fig. 5:**
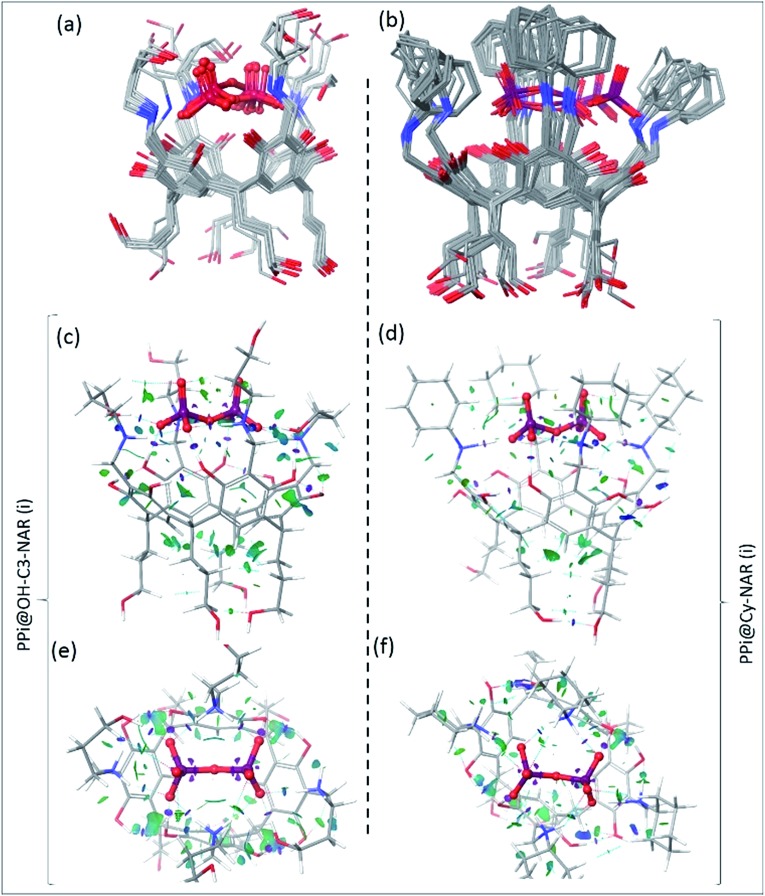
The superimposed conformers of (a) PPi@OH-C3-NAR within 5 kcal mol^–1^ and (b) PPi@Cy-NAR within 15 kcal mol^–1^ of the global minimum calculated using the OPLS-2005 force field; note that despite this wide energy range, the PPi and H_2_N^+^ of receptor are mostly fixed in space, with the greatest movement observed for the upper hydroxyls not directly involved in the binding interaction. B3LYP/6-31G** optimized conformers and calculated NCI gradient isosurfaces with *s* = 0.3 au representing the intra-host and host–guest non-covalent interactions in (c, e) PPi@OH-C3-NAR and (d, f) PPi@Cy-NAR, side view and top view. The surfaces are colored based on the values of sign(*λ*_2_)*ρ* between –0.5 to 0.5 au. In this colouring system, green and yellow isosurfaces correspond to weakly attractive and weakly repulsive interactions respectively, whereas blue boundaries indicate strongly attractive interactions, and red strongly repulsive. Note the lack of repulsive interactions in these systems. The very strong attractive intra-host and host–guest H-bonds (*i.e.* O–H···O–H bonds) are shown as disks of purple colour, and significant non-covalent bond paths indicated with dashed blue lines with bond critical points in the density.

To determine the preferred binding mode of both the PPi@OH-C3-NAR and the PPi@Cy-NAR complexes, a thorough conformational analysis was performed using the MacroModel/Maestro software package^47^ with the OPLS-2005 force field (see ESI† for details). This provided a series of related low energy conformations and binding modes of the host–guest complexes within 5 or 15 kcal mol^–1^ of the global minimum for the two PPi@NAR systems (superimposed conformers; Fig. 5a and b).

PPi@OH-C3-NAR adopts two major related forms, a partially folded-arm conformer, where two of the upper hydroxyl groups fold back over the encapsulated PPi; and a tight folded-arm conformer, where three hydroxyl groups fold back over the PPi. In the former (Fig. 5a, PPi@OH-C3-NAR (i)) the two folded hydroxyl groups form strong H-bonds with the PPi anion. These interactions are supplemented by additional charge-assisted and neutral H-bonds^48^ as well as the other types of noncovalent interactions^49^ that strengthen the host–guest interaction. In the latter (Fig. S18,† PPi@OH-C3-NAR (ii)) three of the hydroxyl groups interact with the PPi, together with a series of intramolecular and intermolecular attractive interactions to better organize the complex and improve binding affinity. These two conformations account for around 70% of the low energy conformations. No other cluster accounts for more than 15% of the remaining low energy structures (see ESI, Fig. S18†). PPi@Cy-NAR demonstrates a narrower conformational range than PPi@OH-C3-NAR; a smaller number of low-energy conformers were found to be within 15 kcal mol^–1^ above the global minimum with lower conformational diversity. This highlights the more rigid structure of this complex featuring cyclohexyl groups in the upper rim. The families of low energy conformers are very similar and are represented in Fig. 5b (PPi@Cy-NAR(i) and Fig. S19†); unlike for PPi@OH-C3-NAR, the lack of upper rim hydroxyl groups limits the number and strength of attractive interactions, and the differences between the different families are most apparent in the change in angle of the cyclohexyl groups.

To better investigate the water-solvated ternary complexes, we performed density functional theory (DFT) optimization on representative low energy conformers of both complexes at the (B3LYP/6-31G**) level of theory. Solvent effects in water were accounted for using the standard Poisson–Boltzmann Finite-Element (PBF) implicit solvation model, using a dielectric constant of 80.37 and probe radius of 1.4 Å as implemented in the Jaguar suite of programs (Schrodinger version 7.6).^50^

To better describe, quantify, and visualize the inter- and intramolecular interactions responsible for the formation of the preferred conformations, we carried out a noncovalent interaction (NCI)^51^ analysis on representative DFT-derived optimized conformations. These calculations provide a quantitative analysis of each and every interaction between unbound atoms and allow for an in-depth analysis of the key noncovalent interactions; a fuller description of the mathematics involved is provided in the ESI,† but in simple terms, the magnitude of attractive or repulsive interactions is a function of electron density and its gradient. If [sign(*λ*_2_)*ρ*] < 0, then the force is attractive, while if [sign(*λ*_2_)*ρ*] > 0, the force is repulsive.

Fig. 5c–f shows the NCI isosurfaces of complexes PPi@OH-C3-NAR and PPi@Cy-NAR. There are few if any repulsive interactions. Complex PPi@OH-C3-NAR shows considerably more and stronger noncovalent interactions than complex PPi@Cy-NAR, represented by more and wider NCI surfaces. The strength of the interactions can be quantified using the NCI interaction critical points ((+/–)*ρ*_ICP_): the more negative the density, the stronger the attraction (Table 3). For the intra-host subclass, the homonuclear O–H···OH H-bonds are slightly stronger in PPi@OH-C3-NAR than PPi@Cy-NAR; however, the heteronuclear positive charge assisted H-bonds, N^+^–H···OH, are significantly stronger in complex PPi@OH-C3-NAR. As expected, the biggest difference between the two complexes lies in the strength of host–guest affinity. Both charge-assisted intermolecular H-bonds, N^+^–H···^–^O–P and O–H···^–^O–P, are significantly stronger in PPi@OH-C3-NAR than PPi@Cy-NAR. Both the conformational scan and the NCI analysis strongly suggest a higher binding affinity of PPi for PPi@OH-C3-NAR over PPi@Cy-NAR. This was further confirmed by our DFT based (B3LYP/6-31G**) optimization of PPi@OH-C3-NAR and PPi@Cy-NAR and calculation of interaction energies (Δ*E*_int_) of –131 *vs.* –62 kcal mol^–1^ for PPi@OH-C3-NAR and PPi@Cy-NAR, respectively (see ESI†).

**Table 3:** NCI interaction critical points ((+/–)*ρ*_ICP_) representing the intra-host and host–guest noncovalent interactions in complex PPi@OH-C3-NAR, PPi@Cy-NAR

Complex	Noncovalent interactions	(–)*ρ*_ICP_	Complex	Noncovalent interactions	(–)*ρ*_ICP_
	Intra-host^*a*^			Intra-host^*a*^	
PPi@OH-C3-NAR	O–H···OH	–0.0343	PPi@Cy-NAR	O–H···OH	–0.0341
	O–H···OH	–0.0153		O–H···OH	–0.0151
	N^+^–H···OH	–0.0286		N^+^–H···OH	–0.0170
	**Total**	**–0.0782**		**Total**	**–0.0662**

Complex	Noncovalent interactions	(–)*ρ*_ICP_	Complex	Noncovalent interactions	(–)*ρ*_ICP_
	Host–guest^*a*^			Host–guest^*a*^	
PPi@OH-C3-NAR	O–H···^–^O–P (5)	–0.0454	PPi@Cy-NAR	O–H···^–^O–P (2)	–0.0398

^*a*^The high densities (*i.e.*, 0.005 < *ρ*(*r*) < 0.05 au) associated with these NCI interactions are consistent with their strong noncovalent character and indicative of attractive bonding interactions.

Our computational analysis strongly supports the enhanced binding of PPi to OH-C3-NARCl over Cy-NARCl. This is due to a combination of factors including: (1) the tighter complex of PPi@OH-C3-NAR, (2) the formation of additional H-bonds between host and guest in PPi@OH-C3-NARCl due to the presence of the upper-rim hydroxyls, and (3) the stronger and larger number of other types of noncovalent interactions; together these factors all lead to an improved fit and complementarity between the anionic PPi and the cationic cavitand in PPi@OH-C3-NARCl rather than PPi@Cy-NAR.

## Conclusions

In summary, a comprehensive solution study using ^1^H and ^31^P NMR, UV-vis and ITC confirms the high-affinity binding of PPi in H_2_O by the NARCl receptors. Host-guest binding of the phosphates (PO_4_^3–^, PPi and ATP) was confirmed by ^1^H and ^31^P NMR experiments in a mainly 1 : 1 binding stoichiometry observed from Job plot experiments. These complexes were also observed in the gas phase through ESI-MS. The interaction between the NARCl receptors and the phosphates was supported *via* UV-vis experiments. Quantification of the binding process through a series of ITC experiments reveal a much higher affinity for PPi over ATP. The receptor with the terminal propyl hydroxyl groups at the upper rim OH-C3-NARCl, gave an extremely high binding constant (PPi@OH-C3-NAR: *K*_1_ = 1.60 ± 0.77 × 10^7^ M^–1^) for PPi. This high binding constant is two orders of magnitude higher than for the less flexible ethyl hydroxyl host and three orders of magnitude higher than any other host–phosphate ensemble (for example ATP@OH-C3-NAR: *K*_1_ = 1.22 ± 0.36 × 10^4^ M^–1^). Guest displacement assay experiments using both ^1^H NMR and UV-vis analyses with 2-naphthalenesulfonic acid sodium salt further support OH-C3-NARCl selectivity for PPi. The preferential binding for PPi was quantified *via* a thorough conformational search and density functional theory (DFT) study that highlighted the significance of the key multiple attractive noncovalent interactions as important factors for establishing the high binding affinity. Associated with these enhanced attractive binding interactions in the favored structure was the presence of complementarity, which led to the enhanced binding between the cationic host and the anionic guests. These results clearly show that the NARCl receptors are both extremely selective and potent systems for the detection of PPi in water through high-affinity binding. The binding affinity was much lower in a Tris buffer suggesting interference of the ionic buffer with the ionic receptor and ionic guests as would be expected, but demonstrates that the NMR and ITC observations are not due to simple protonation/deprotonation processes but rather to true host–guest interactions. Considering that the NARX receptors were shown to be selective for chlorides over other anions (bromide, iodide, nitrate, triflate and picrate),^33^ the binding constants for this system can be further increased as needed. It is also expected that the detailed rationale offered here will assist researchers in designing additional novel host–guest complexes incorporating various complementary attractive noncovalent interactions as key factors for promoting binding processes. These results clearly demonstrate the excellent binding capability and selectivity of this system, and we are currently modifying the receptors to provide additional functionality to broaden the analytical techniques for detection in biological systems beyond those described here, as well as study how different buffers affects the binding affinity of these ionic species.

## Conflicts of interest

The authors declare no conflicts of interest.

## Supplementary Material

Supplementary information
