# Supplementary material for: High-affinity and selective detection of pyrophosphate in water by a resorcinarene salt receptor
Source: Chem Sci. 2017 Dec 19;9(5):1358–67. doi: 10.1039/c7sc05167k (PMC5887233; doi:10.1039/c7sc05167k)
Supplement: Supplementary file 1 [file SC-009-C7SC05167K-s001.pdf]

Electronic Supplementary Material (ESI) for Chemical Science.
This journal is © The Royal Society of Chemistry 2017

High-Affinity and Selective Detection of Pyrophosphate in Water by a Resorcinarene Salt Receptor

Ngong Kodiah Beyeh,*[a,b] Isabel Díez,[a] S. Maryamdokht Taimoory,[b] Daniel Meister,[b] Andrew Feig,[d] John F. Trant,*[b] Robin H. A. Ras,*[a,c] and Kari Rissanen*[e]

[a] Aalto University, School of Science, Department of Applied Physics, Puumiehenkuja 2, FI-02150, Espoo, Finland
[b] University of Windsor, Department of Chemistry and Biochemistry, Windsor, ON N9B 3P4, Canada
[c] Aalto University, School of Chemical Engineering, Department of Bioproducts and Biosystems, Kemistintie 1, 02150 Espoo, Finland
[d] Wayne State University, Department of Chemistry, 5101 Cass Ave., Detroit, MI, 48202, USA
[e] University of Jyvaskyla, Department of Chemistry, Nanoscience Center, P. O. Box 35, FI-40014 Jyväskylä, Finland

Supporting Information

Table of Contents

1 General Information....2
2 Synthesis....2
3 NMR Spectroscopy....3
4 Isothermal Titration Calorimetry (ITC) in H_2O....6
5 Competition Experiments – NMR....7
6 Mass Spectrometry....8
7 UV-Vis/Fluorescence Spectroscopy....9
8 pH Measurements....11
9 Isothermal Titration Calorimetry (ITC) in TRIS Buffer....11
10 Computational Modelling....12
11 References....16

1 General Information

The *N*-alkyl ammonium resorcinarene chlorides (NARCls) were synthesized according to reported procedures.[1-4] The phosphates (K_3PO_4, Na_4PPi, Na_2ATP), and 2-naphthalenesulfonic acid sodium salt (NSANa) were purchased from commercial sources and used as such. The synthesis of OH-C2-NARCl and Cy-NARCl has been reported elsewhere.[4] ^{1}H and ^{13}C NMR spectra were recorded on a Bruker Avance DRX 500 (500 MHz for ^{1}H and 126 MHz for ^{13}C) and Bruker Avance DRX 400 (400 MHz for ^{1}H and 161.89 MHz for ^{31}P) spectrometers. All signals are given as δ values in ppm using residual solvent signals as the internal standard. Coupling constants are given in Hz. Melting points were determined with a Mettler Toledo FP62 capillary melting point apparatus. Experimental details for the synthesis and characterization data of OH-C3-NARCl is below.

2 Synthesis

Synthetic details of *N*-propanol ammonium resorcinarene chloride (OH-C3-NARCl)

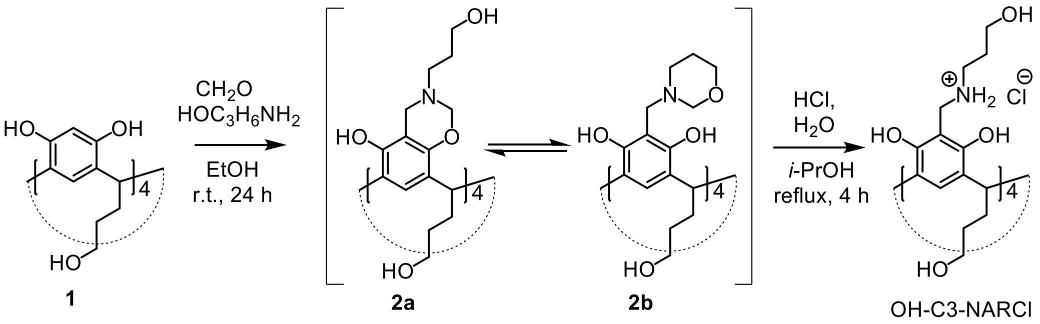

Scheme S1. Synthesis of *N*-propanol ammonium resorcinarene chloride (OH-C3-NARCl).

To a solution of the resorcinarene **1** (2 g, 2.77 mmols) and excess formaldehyde (4 mL) in ethanol (40 mL), 3-aminopropan-1-ol (0.88 mL, 11.6 mmols) in ethanol (15 mL) was added slowly and stirred at room temperature for 24 h. The precipitate that separated was filtered, recrystallized in a methanol/n-hexane mixture and dried. The product was a mixture of two six-membered ring tetrabenzoxazines **2a/2b**. The mixture of tetrabenzoxazines was not separated. Into a solution of the tetrabenzoxazine (1.0 g 0.89 mmol), 3 mL concentrated HCl (37 %) and 4 ml H_2O in 50 ml isopropanol is heated under reflux. Water and formaldehyde were removed by azeotropic distillation with chloroform. The remaining isopropanol was evaporated and the crude product recrystallized from $CHCl_3$-MeOH. After filtration with CH_2Cl_2/MeOH 10%, the *N*-propanol ammonium resorcinarene chloride OH-C3-NARCl (1.98 g, 58 %) was isolated. m.p. > 300 °C; ESI-TOF-HRMS (Positive ion mode, sprayed from MeOH): *m/z* Found 1069.5959 $[M-4Cl-3H]^+$, (-0.4 mDa, -0.4 ppm); calc. 1069.5955. ^{1}H NMR (500 MHz, 303 K in D_2O) δ (ppm): 1.45 (m, 8H, CH_2), 1.74 (m, 8H, CH_2), 2.23 (m, 8H, CH_2), 2.96-3.06 (m, 8H, NCH_2), 3.48-3.67 (m, 16H, OCH_2), 4.18 (s, 8H, $ArCH_2N$), 4.37 (t, J=7.50 Hz, 4H, CH), 7.32 (s, 4H, ArH); ^{13}C NMR: (100 MHz, 303 K in D_2O) δ (ppm) = 27.5, 28.9, 29.7, 34.4, 37.3, 41.5, 44.7, 48.8, 59.1, 61.6, 108.9, 125.0, 126.6, 150.9.

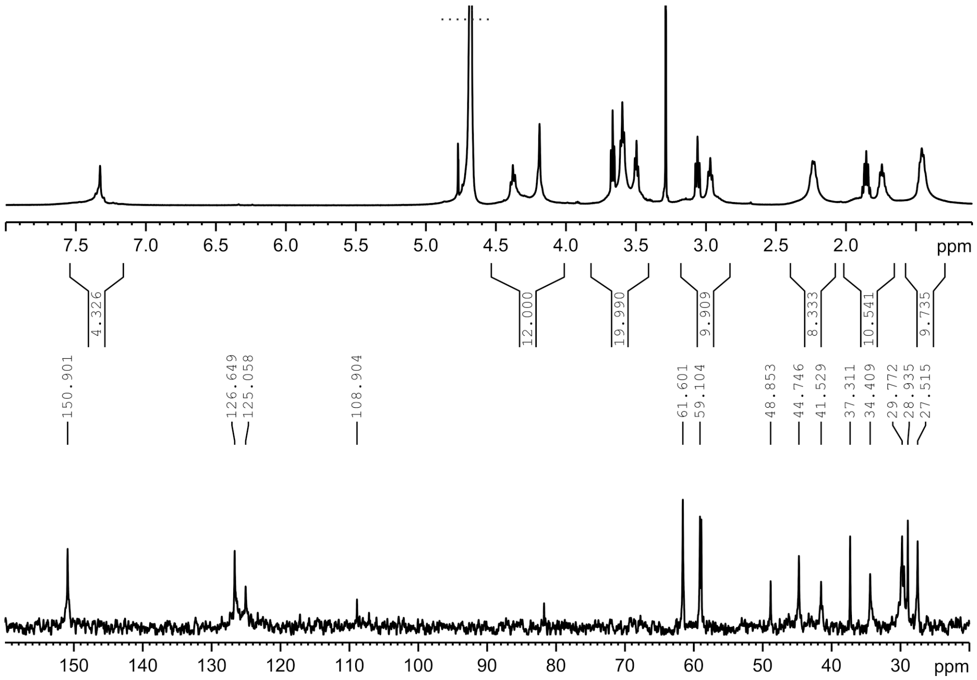

Figure S1. ^{1}H and ^{13}C NMR spectra of *N*-propanol ammonium resorcinarene chloride OH-C3-NARCl in D_2O at 303 K.

3 NMR Spectroscopy

For sample preparation, 5 mM stock solutions of the receptors (OH-C3-NARCl and Cy-NARCl), the phosphates (K_3PO_4, Na_4PPi, Na_2ATP), and the guest NSANa were prepared. For the pure receptors and phosphates guests, 250 µL of the stock solution was measured to NMR tube and diluted with 250 µL of pure D_2O to give a 2.5 mM sample concentration. For a 1:1 host-guest (receptor-phosphate) mixtures, 250 µL of the host and 250 µL of the guests were measured to give a 2.5 mM concentration of both the host and the guests. The spectra were calibrated using D_2O signal as internal standard. For ^{31}P NMR, H_3PO_4 in a 2mm NMR tube was inserted into the sample as an external calibrant, and calibrated as 0 ppm. Samples without H_3PO_4 as external calibrant were also measured. In the samples containing K_3PO_4, H_3PO_4 could not be used as an external standard due to overlapping of the PO_4 signals.

a) ^{1}H NMR

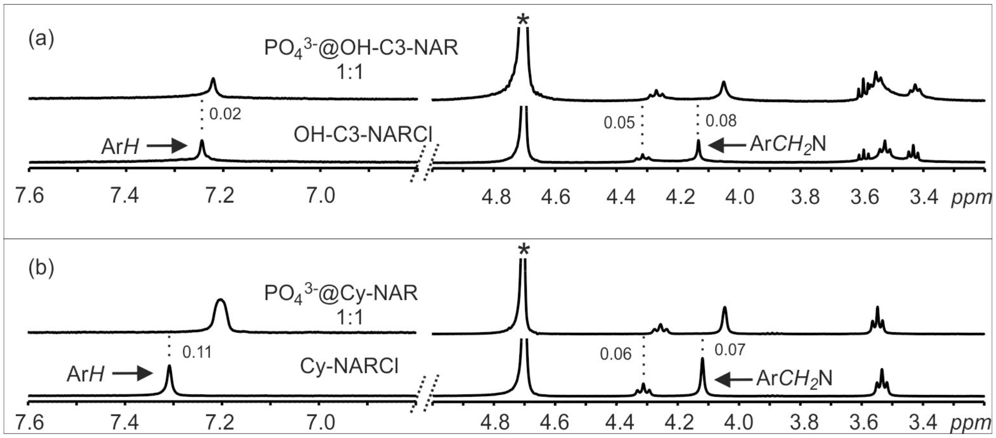

Figure S2. ^{1}H NMR spectra (D_2O, 298 K) of: (a) OH-C3-NARCl and the equimolar mixture OH-C3-NARCl and PO_4^{3-}, (b) Cy-NARCl and the equimolar mixture Cy-NARCl and PO_4^{3-}. Star represents the residual D_2O solvent. The dash lines give an indication of the signal changes in ppm.

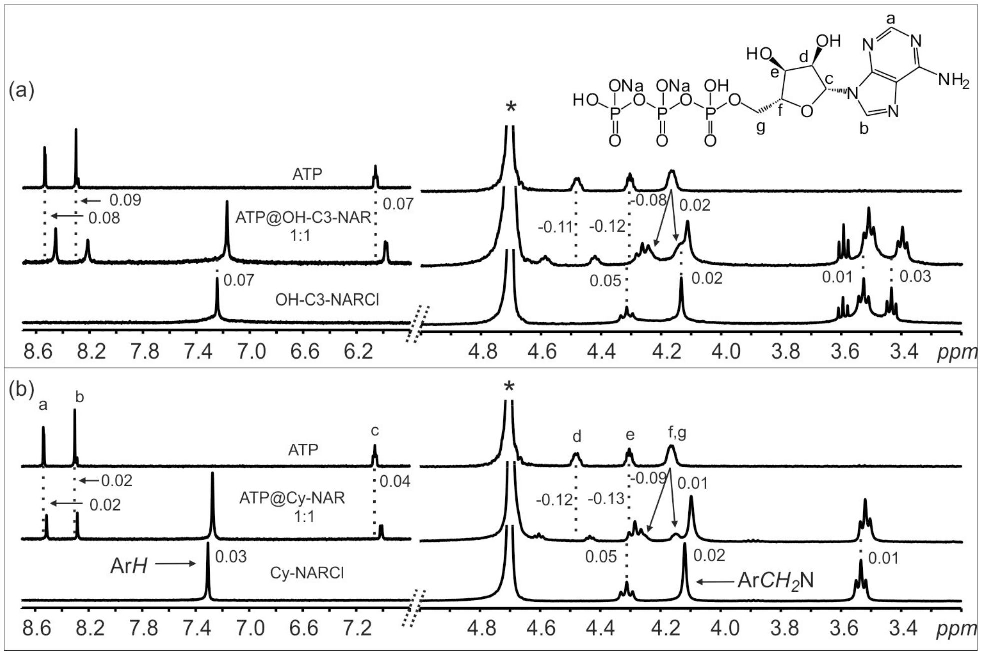

Figure S3. ^{1}H NMR spectra (D_2O, 298 K) of: (a) OH-C3-NARCl, ATP and the equimolar mixture OH-C3-NARCl and ATP, (b) Cy-NARCl, ATP and the equimolar mixture Cy-NARCl and ATP. Star represent the residual D_2O solvent. The dash lines give an indication of the signal changes in ppm.

b) ^{31}P NMR

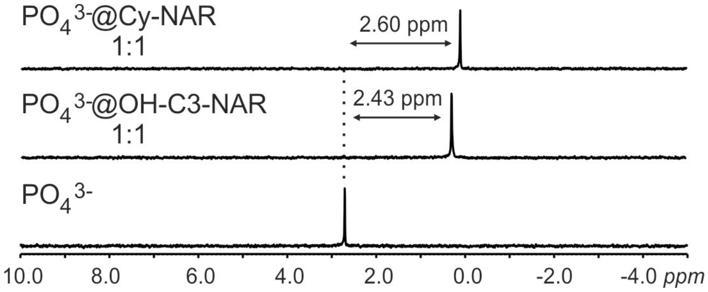

Figure S4. ^{31}P NMR spectra (D_2O, 298 K) of K_3PO_4, and equimolar mixtures of OH-C3-NARCl and PO_4^{3-} and Cy-NARCl and PO_4^{3-}. The dash lines give an indication of the signal changes in ppm.

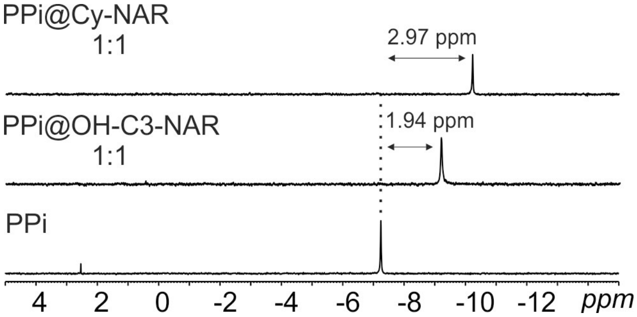

Figure S5. ^{31}P NMR spectra (D_2O, 298 K) of PPi, and equimolar mixtures of OH-C3-NARCl and PPi and Cy-NARCl and PPi. The dash lines give an indication of the signal changes in ppm.

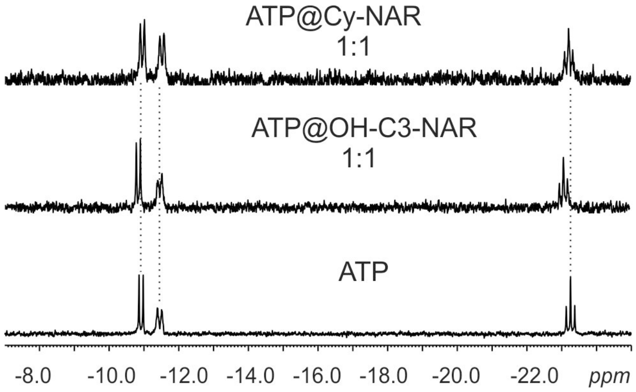

***Figure S6**.* ^{31}P NMR spectra (D_2O, 298 K) of ATP, and equimolar mixtures of OH-C3-NARCl and ATP and Cy-NARCl and ATP.

c) Job Plot Experiments

For Job plot[5] measurements, 5 mM stock solutions of the NARCl receptors, and the phosphate guests (K_3PO_4, Na_4PPi, Na_2ATP) were prepared in D_2O. 11 samples of varying equivalents of NARCls and phosphates were prepared (NARCl + phosphates v/v mL: 0.50+0.00, 0.45+0.05, 0.40+0.10, 0.35+0.15, 0.30+0.20, 0.25+0.25, 0.20+0.30, 0.15+0.35, 0.10+0.40, 0.05+0.45, 0.00+0.50). The spectra were calibrated using D_2O signal as internal standard.

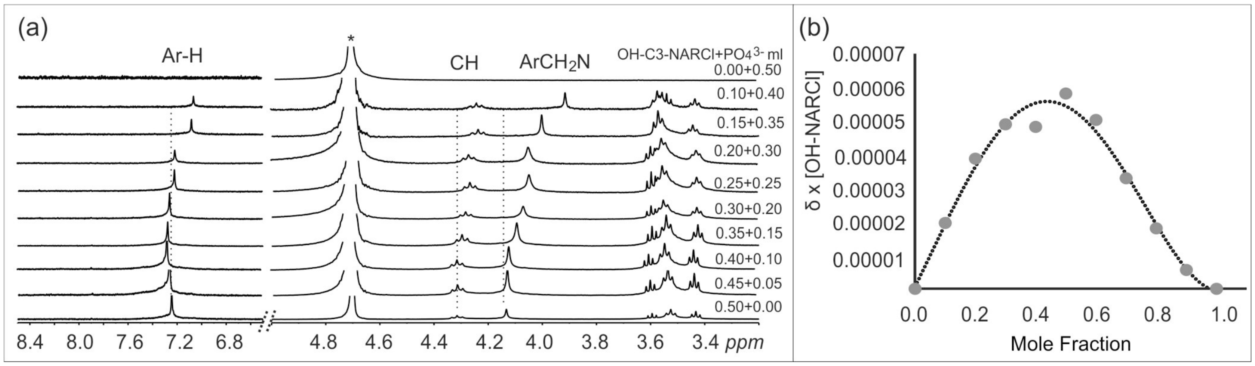

***Figure S7**.* (a) ^{1}H NMR spectra (D_2O, 298 K) of different equivalents of K_3PO_4 to OH-C3-NARCl. The dash lines give an indication of the signal changes presented in ppm. Star represents the residual D_2O solvent. (b) Job plot analysis of OH-C3-NARCl and PO_4^{3-} mixture revealing a 1:1 binding stoichiometry.

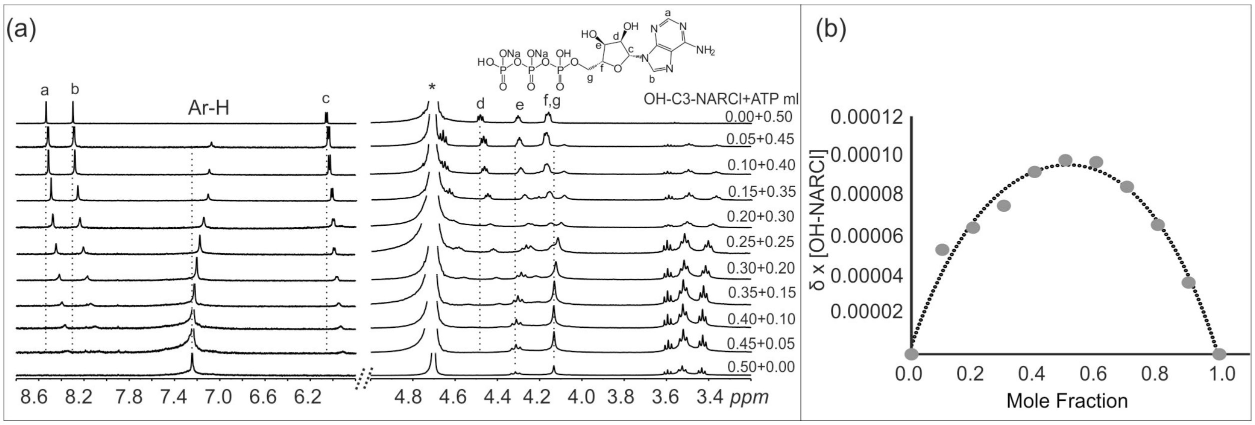

***Figure S8**.* (a) ^{1}H NMR spectra (D_2O, 298 K) of different equivalents of ATP to OH-C3-NARCl. The dash lines give an indication of the signal changes presented in ppm. Star represents the residual D_2O solvent. (b) Job plot analysis of OH-C3-NARCl and ATP mixture revealing a 1:1 binding stoichiometry.

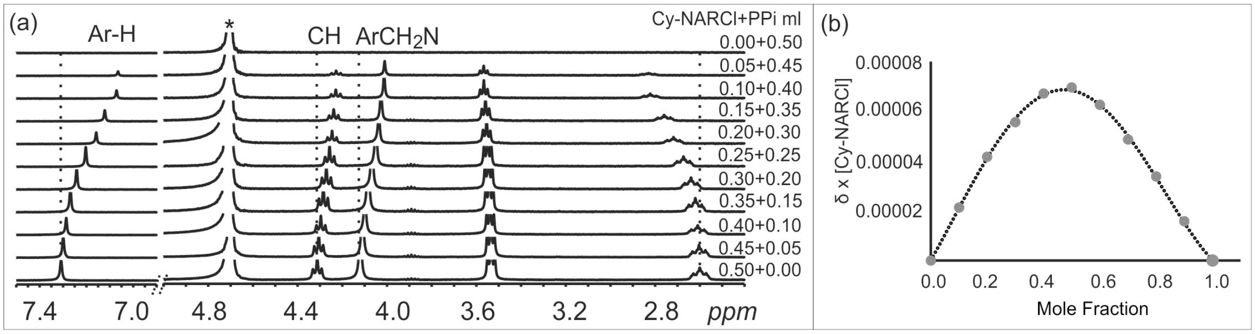

Figure S9. (a) ^{1}H NMR spectra (D_2O, 298 K) of different equivalents of PPi to Cy-NARCl. The dash lines give an indication of the signal changes presented in ppm. Star represents the residual D_2O solvent. (b) Job plot analysis of Cy-NARCl and PPi mixture revealing a 1:1 binding stoichiometry.

4 Isothermal Titration Calorimetry (ITC) Measurements in H_2O

VP-ITC instrument made by MicroCal was used to determine the molar enthalpy (ΔH) of complexation. Subsequent fitting of the data to a 1:1 binding model using Origin software provides binding constant (K), change in enthalpy (ΔH) and entropy (ΔS). For the titration of PPi to OH-C3-NARCl, the data was fitted to a two-sites binding model. The ITC experiment was carried out by filling the sample cell with the substrate solution (0.25 mM), filling the syringe with the titrant (5 mM), and titrating via computer-automated injector at 303 K. Blank titrations into plain solvent were also performed and subtracted from the corresponding titration to remove any effect from the heats of dilution from the titrant.

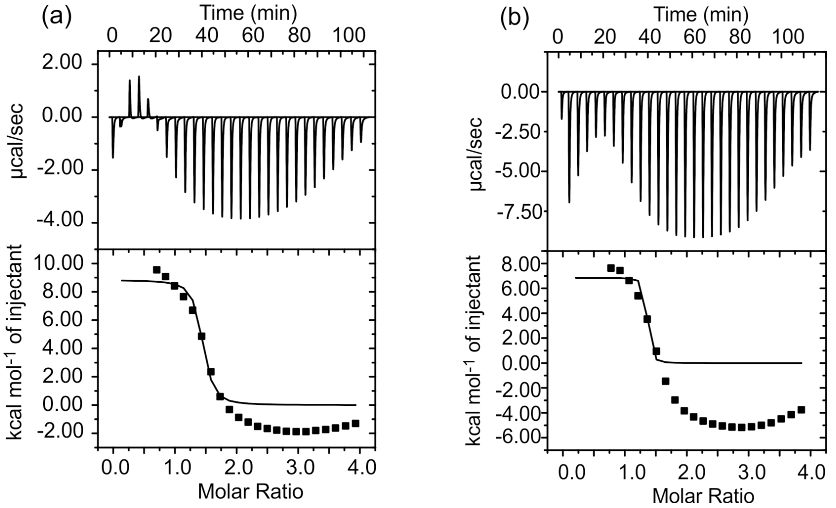

Figure S10. ITC traces of the titration of receptors with K_3PO_4 in H_2O at 303 K. The data could not be fitted to any model: (a) with OH-C3-NARCl, (b) with Cy-NARCl.

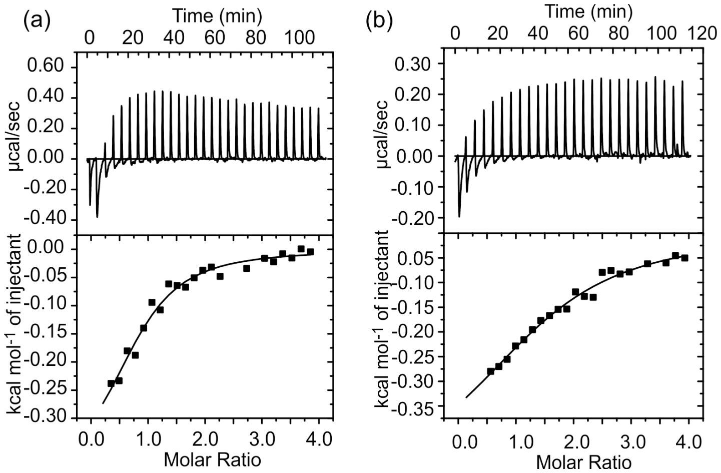

Figure S11. ITC traces of the titration of receptors with ATP in H_2O at 303 K. The data was fitted into a one-site binding model: (a) with OH-C3-NARCl, (b) with Cy-NARCl.

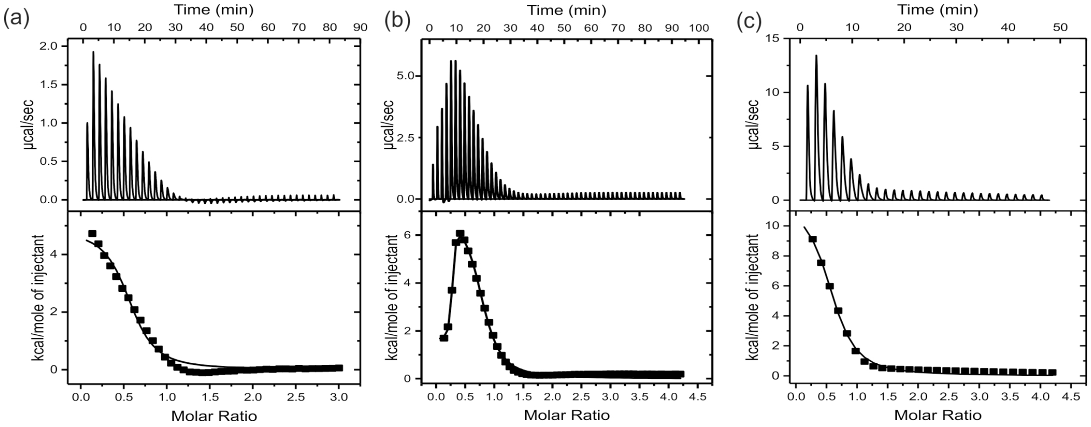

Figure S12. ITC traces of the titration of receptors with PPi in H_2O at 303 K. The data for OH-C2-NARCl and Cy-NARCl was fitted into a one-site binding model while that for OH-C3-NARCl was fitted into a two-site binding model: (a) with OH-C2-NARCl, (b) with OH-C3-NARCl and (c) with Cy-NARCl.

5 Competition Experiments – NMR

Simple ^{1}H NMR competition experiments between PPi and ATP were done to show the preference for PPi. In the experiments, to an equimolar mixture of OH-NARCl and PPi or ATP, one equivalent of the other guest was added and the NMR was recorded. The results were then compared to when no competing guest is present.

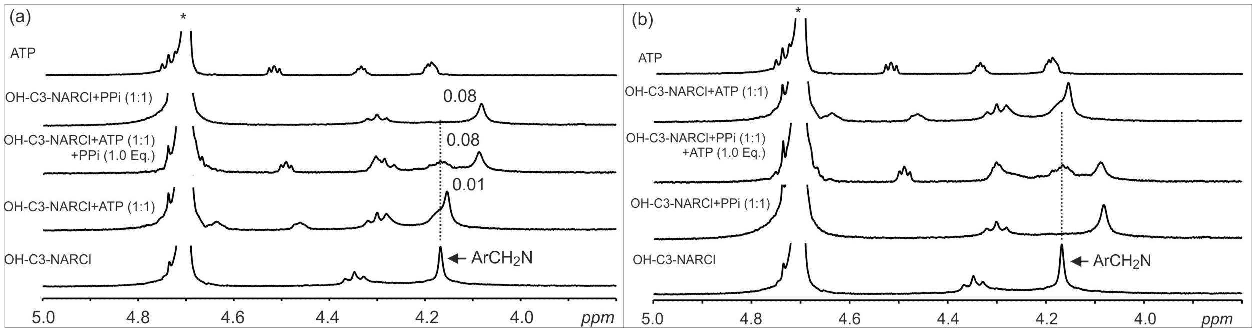

Figure S13. ^{1}H NMR spectra (D_2O, 298 K) of competition experiment between OH-C3-NARCl towards ATP and PPi: (a) Results show PPi to displace ATP, (b) the addition of ATP did not affect the spectrum since ATP cannot displace PPi.

6 Mass Spectrometry

The mass spectrometric studies were performed with a Bruker amaZon speed ETD mass spectrometer with an electrospray ionization (ESI) source and an iontrap detector/analyser. All experiments were performed on positive polarization. Experiments with PPi were also performed on negative polarization. The parameters of the ion source, capillary voltage were optimized to get maximum abundance of the ions under study.

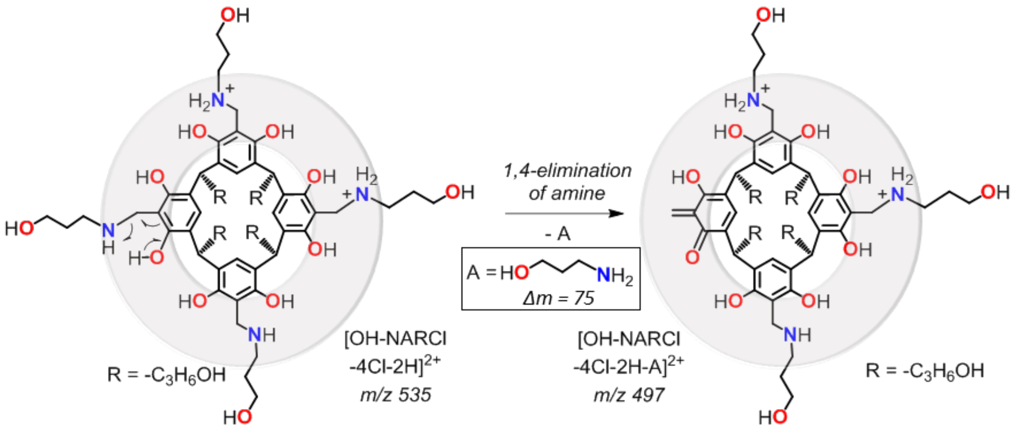

Scheme S2. Fragmentation mechanism and pathway for the 1,4-elimination of amine (A) from [OH-C3-NARCl-4Cl-2H]$^{2+}$.

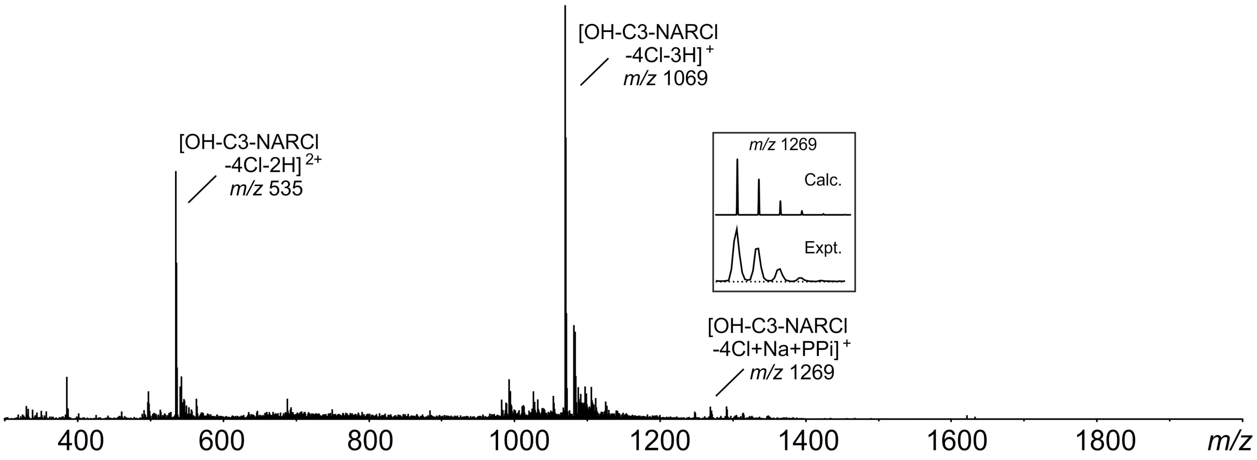

Figure S14. (a) Positive ESI mass spectrum of an equimolar mixture (20 µM) of OH-C3-NARCl and Na_4PPi. Insets: experimental and calculated isotope patterns of selected signals.

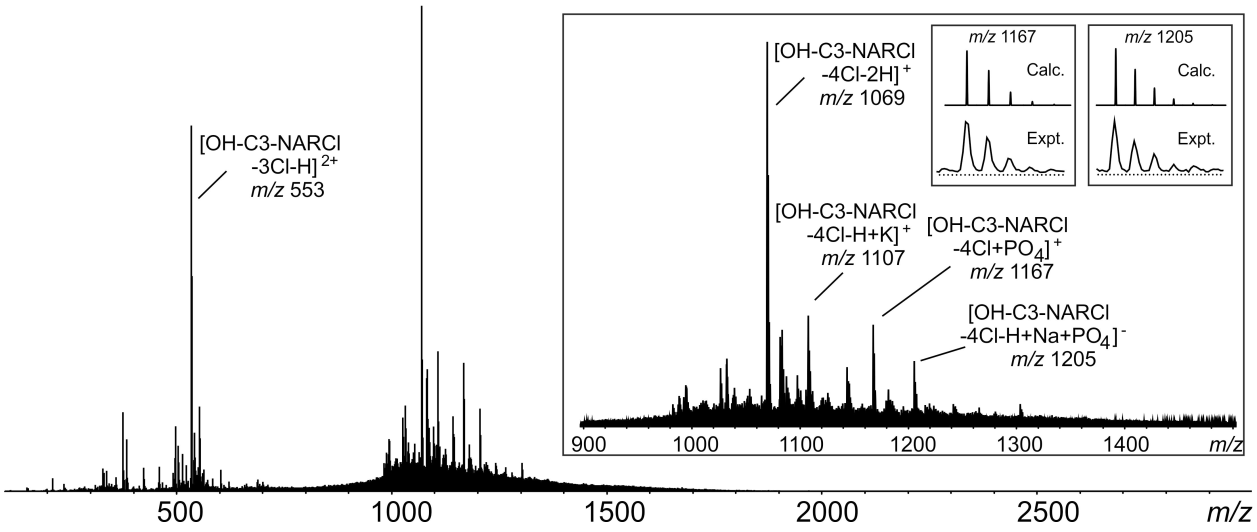

Figure S15. (a) Positive ESI mass spectrum of an equimolar mixture (20 μM) of OH-C3-NARCl and K_3PO_4. Insets: experimental and calculated isotope patterns of selected signals

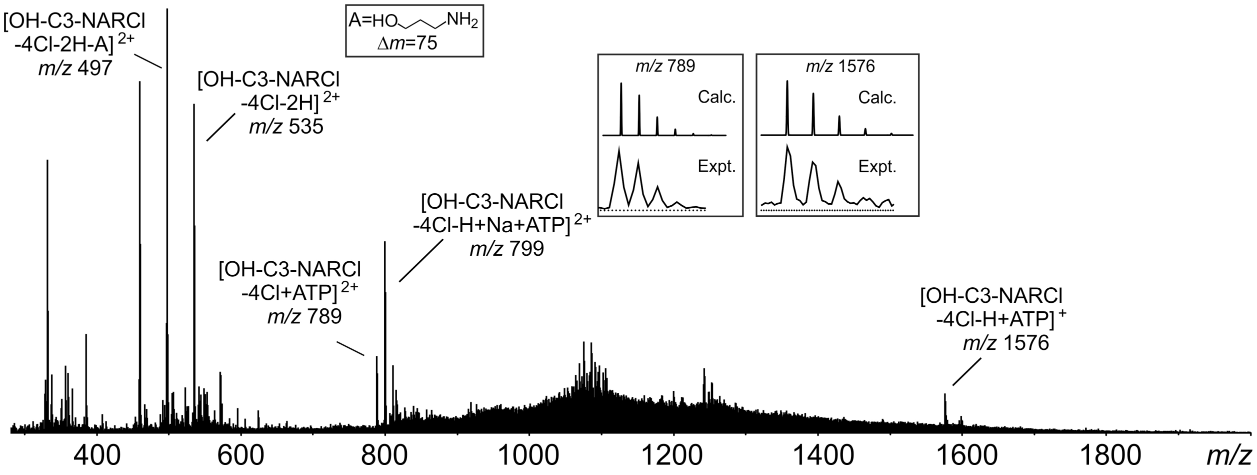

Figure S16. (a) Positive ESI mass spectrum of an equimolar mixture (20 μM) of OH-C3-NARCl and Na_2ATP. Insets: experimental and calculated isotope patterns of selected signals

7 UV-Vis/Fluorescence Spectroscopy

Optical absorption spectra of the solutions were recorded using a PerkinElmer Lambda 950 UV/Vis/NIR spectrophotometer. Fluorescence spectra were obtained with a Quantamaster 40 (Photon Technology International) fluorescence spectrometer. The excitation and emission slits were set to 5 nm and an instrument correction function supplied by the manufacturer was applied to the data. No filters were used in excitation or emission channels to avoid filter-based artefacts. Emission spectra were recorded at the excitation of 500 nm and excitation spectra were recorded at the emission of 525 nm. All absorption and fluorescence spectra were recorded with quartz cells of 10 mm optical path length.

The UV-Vis and fluorescence spectroscopic analyses at micromolar concentrations were carried out to gain further insight into binding properties of the OH-C3-NARCl with the phosphates. For this purpose, titrations experiments were carried with the phosphates as the titrant. Different equivalents of the phosphates were titrated to a 20μM solution of the OH-C3-NARCl in water and under stirring. Absorption and emission spectra were recorded 5 mins after preparation. The concentration of the stock titrant solution was as high as 400 μM to avoid strong dilution of the receptor during titration.

Absorption spectra of the titration to OH-C3-NARCl are shown in Figure 18a-c. K_3PO_4 and Na_2ATP do not produce significant changes in the absorption spectra of OH-C3-NARCl after the addition of the first equivalent while Na_4PPi causes a red shift of about 4 nm in the peak centered at about 300 nm.

Emission and excitation spectra of the titration to OH-C3-NARCl are shown in Figure 18d-f. K_3PO_4 does not produce significant changes while Na_4PPi and Na_2ATP clearly show an important decrease in emission intensity with the first equivalent. Moreover, Na_2ATP causes a noticeable broadening in the region around 600 nm.

Both, absorption and emission spectra of OH-C3-NARCl present visible changes with addition of Na_4PPi suggesting a clear affinity between them.

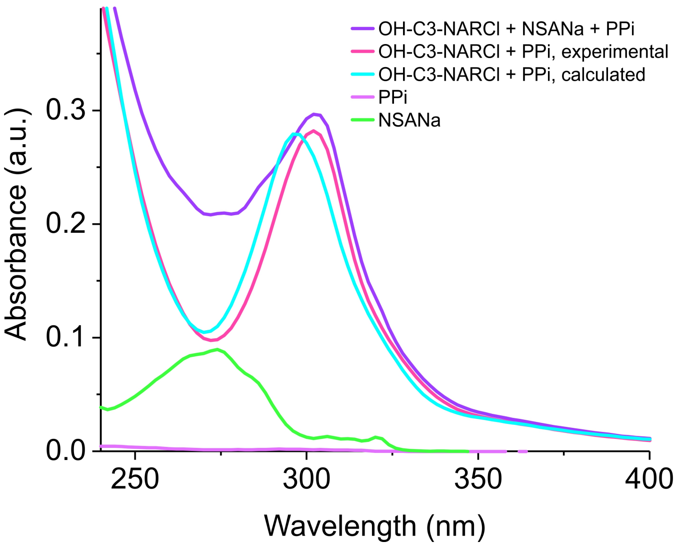

Figure S17. Comparison of the calculated and experimental absorption spectra of PPi@OH-NAR mixture, showing a clear red shift and supporting the idea of strong host-PPi interaction

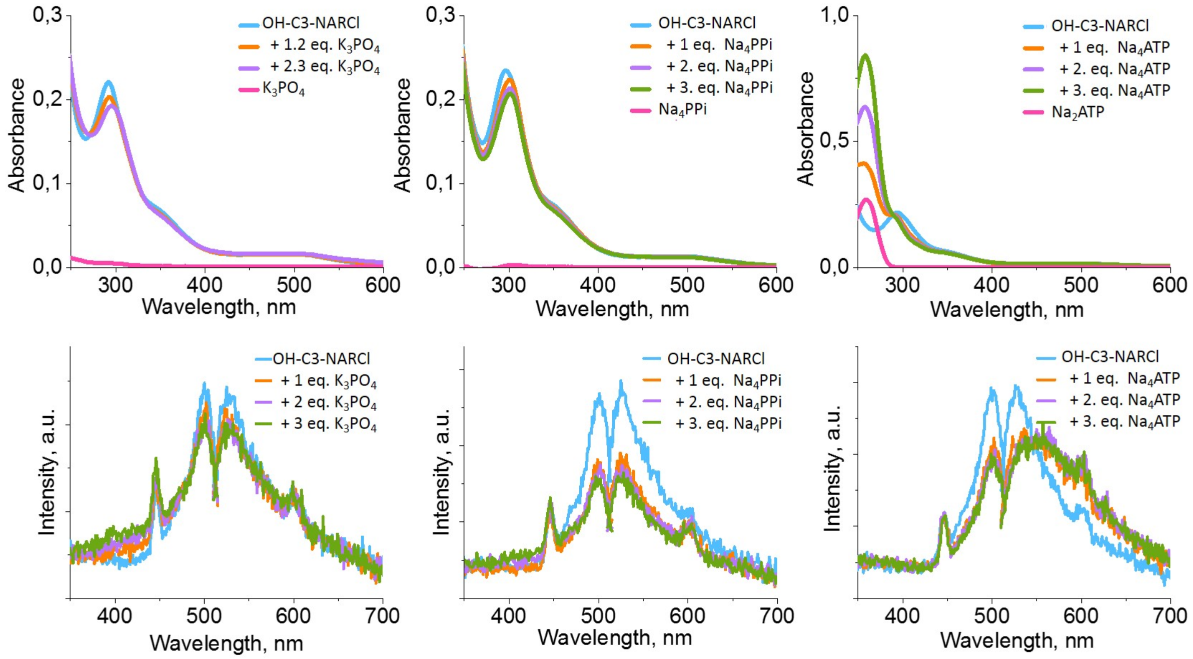

Figure S18. Absorption spectra (a-c) and emission spectra (d-f) of a titration in H_2O to OH-C3-NARCl using as titrants K_3PO_4 (a,d), Na_4PPi (b,e) and Na_2ATP (c,f).

8 pH Measurements

Milli-Q® H_2O was used for all the pH Measurements and allowed to equilibrate under atmosphere to provide a pH of 5.70. The pH meter was calibrated with pH standards on the day of the experiments. The pH of 20 µM solutions of the pure samples and equimolar mixtures (20 µM) were prepared in Milli-Q® H_2O at room temperature which read 18 °C on the day of measurement.

Table S1: pH measurements, 20 µM in H_2O at 18 °C.

Sample	pH	Sample	pH
H_2O	5.70		
OH-C2-NARCl	5.16	Na_3PO_4	6.62
OH-C3-NARCl	4.87	Na_4PPi	6.47
Cy-NARCl	4.67	Na_2ATP	5.37
OH-C2-NARCl + Na_3PO_4	5.91	OH-C3-NARCl + Na_3PO_4	5.80
OH-C2-NARCl + Na_4PPi	5.90	OH-C3-NARCl + Na_4PPi	5.88
OH-C2-NARCl + Na_2ATP	5.97	OH-C3-NARCl + Na_2ATP	5.86
Cy-NARCl + Na_3PO_4	4.73		
Cy-NARCl + Na_4PPi	4.73		
Cy-NARCl + Na_2ATP	4.77		

9 Isothermal Titration Calorimetry (ITC) Measurements in TRIS buffer (pH 7.10)

The titration of OH-C3-NARCl with Na_4PPi was done in 50 mM and 10 mM TRIS buffer at pH 7.10. The data were fitted into a 1:1 binding model using Origin software which provided the thermodynamic parameters. The ITC experiments were performed similarly to section 4 above.

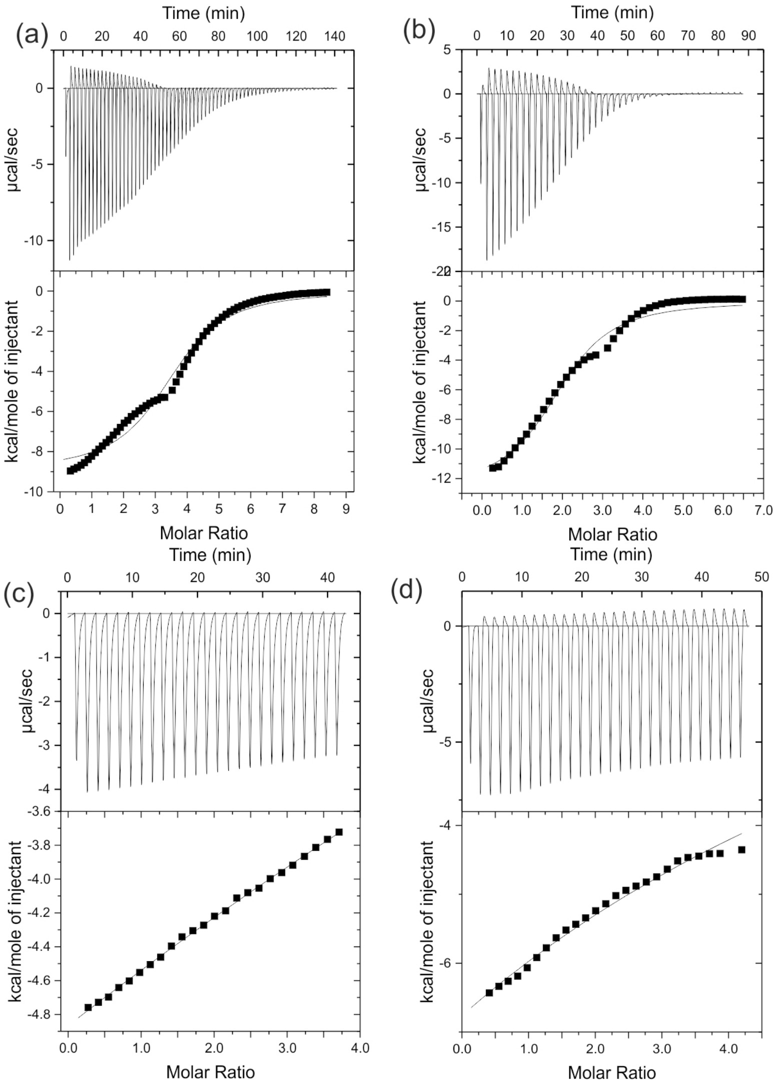

Figure S19. ITC traces of the titration of receptors with PPi in Tris Buffer at pH 7.10 at 303 K. The data were fitted into a one-site binding model. Titrations were done in 10 mM buffer concentration with (a) OH-C3-NARCl and (b) Cy-NARCl. Titrations done in 50 mM buffer concentration with (c) OH-C3-NARCl and (d) Cy-NARCl. The curves clearly show binding between the receptor and PPi guest. The affinity is lower than that observed in pure water as expected due to the competitive nature of the buffer for interacting with both the receptor and the guest. The effect is dose dependent. At 50 mM, the affinity for OH-C3-NARCl is on the order of 10^2, while it is 10^4 with a 10 mM buffer, and 10^7 in pure water as described in the main text.

10 Computational Modelling

The structural modelling was carried out using our previously obtained crystal structures[6] as the initial conformation of the *N*-Alkyl ammonium resorcinarene receptor frameworks. Conformational analysis was performed using MacroModel/Maestro software package[7] with the OPLS-2005 force field in order to probe the shape, rigidity and noncovalent interactions of the PPi@OH-C3-NARCl (**1**) and PPi@Cy-NARCl (**2**) complexes.

Accordingly, the thorough conformational search was performed using the Mixed Torsional/Low-mode (MT/LMOD) algorithm of MacroModel to find all possible conformers of host-guest complexes within a 62.8 kcal/mol energy window of the global minimum structure at ambient temperature. The root mean square deviation (RMSD) cutoff to eliminate redundant conformers was set to 0.25 Å. The generated conformations were then clustered using a clustering of conformers approach implemented in Macromodel to limit the conformers to only those low energy families within 5-15 kcal/mol of the global energy minimum. A representative of each cluster was

then subjected to an energy minimization using the OPLS-2005 force field, with a truncated Newton conjugated gradient (TNCG) method. 5 unique conformers were finally selected for both PPi@OH-C3-NARCl (**1**) and PPi@Cy-NARCl (**2**) complexes and their relative energies were calculated (Figure S2-S3).

A representative low energy conformer of both complexes **1** and **2** were further optimized using density functional theory (DFT) calculations using the hybrid density functional B3LYP with the 6-31G** basis set in water using a PBF implicit solvation model[8] as implemented in the Jaguar suite of programs (Schrodinger version 7.6 and Gaussian 09)[9]. Harmonic vibrational frequencies were also calculated for the optimized complexes.

The noncovalent interactions (NCI) index analysis[10] was carried out on the DFT optimized geometries applying the Schrödinger Jaguar program, and the generated results were visualized with Maestro 11.1. It is important to mention that NCI approach allows us to visualize different classes of noncovalent interactions and evaluate their nature as either repulsive or attractive. This obtain via plotting the magnitude of the electron density signed by the second eigenvalue of the density Hessian, sign(λ_2)ρ, versus regions of very low-reduced density gradient s(r). These isosurfaces highlight both favorable and unfavorable interactions between unbound atoms, defined by the sign of the second Hessian eigenvalue of the density (λ_2) and the map coloring identifies the strength as well as the nature of the interactions. Thus, the values of ρ(r), NCI interaction critical points ((+/-)ρICP), help us to evaluate the strength of interaction and the sign of (λ_2)ρ, help in estimation of the different types of weak interactions. Accordingly, the larger the absolute values of (-)ρICP related with density accumulation (sign(λ_2)ρ < 0), the stronger the detected stabilizing non-covalent interaction.

The calculated interaction energies (Scheme S3) refer to the interaction energy between the receptor and the PPi in the geometry of complex and is calculated according to equation S1 and S2: **1** and **2** are the host-guest assemblies, OH-C3-NARCl and Cy-NARCl are the receptor alone with the same geometry as they have in **1** and **2** complex, and the PPi ligand has the same conformation as it is in **1** and **2**.

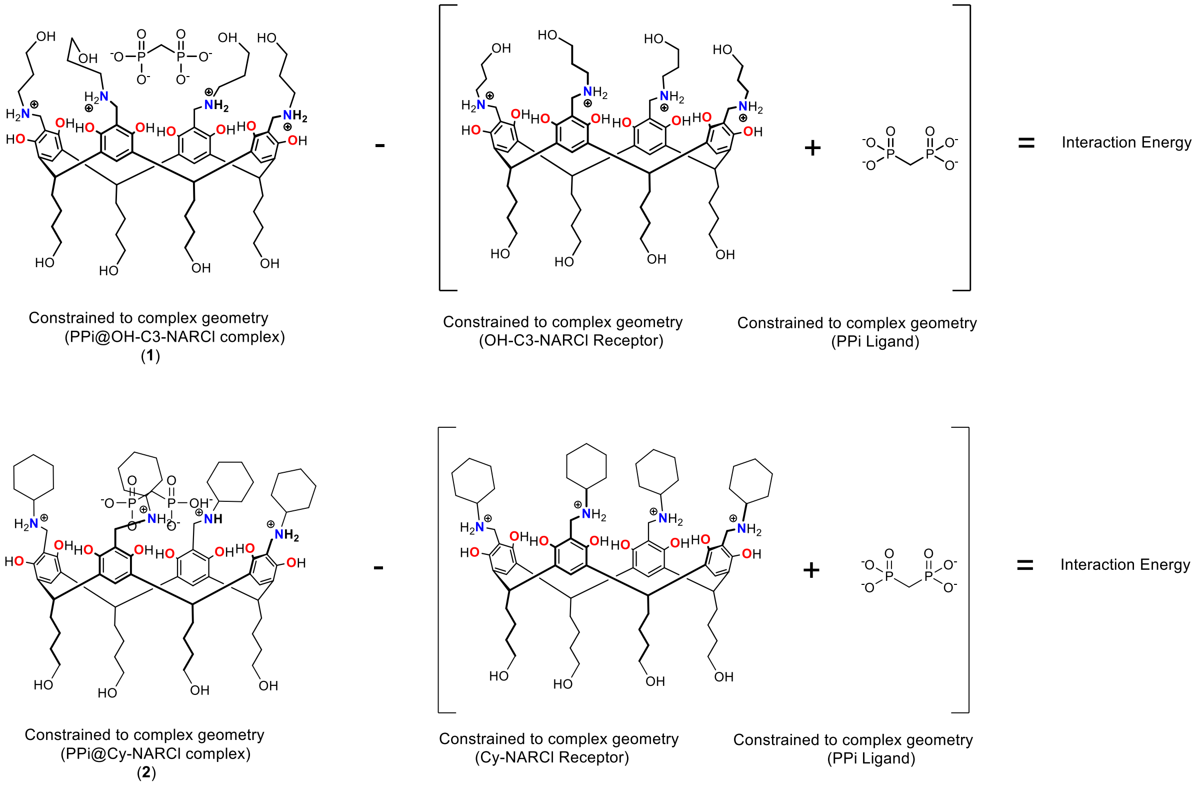

Scheme S3. The calculated interaction energies and the proposed reaction scheme for the PPi@OH-NARCl and PPi@Cy-NARCl refer to the interaction energy between the receptor and the PPi.

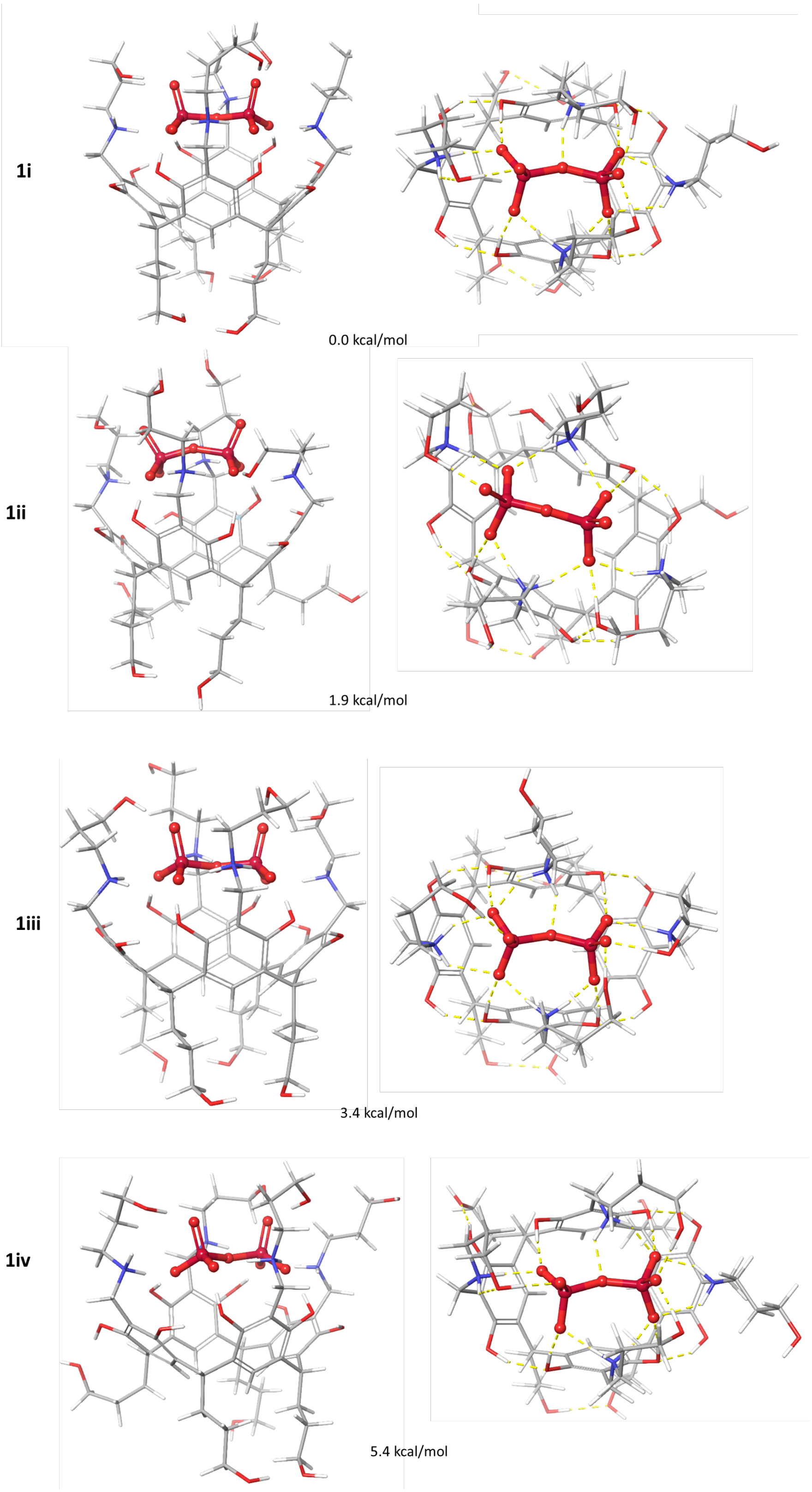

***Figure S20*.** The low energy conformers of PPi@OH-C3-NAR (**1i-1iv**): side view and top view of same, within approximately 5kcal/mol of the global minimum.

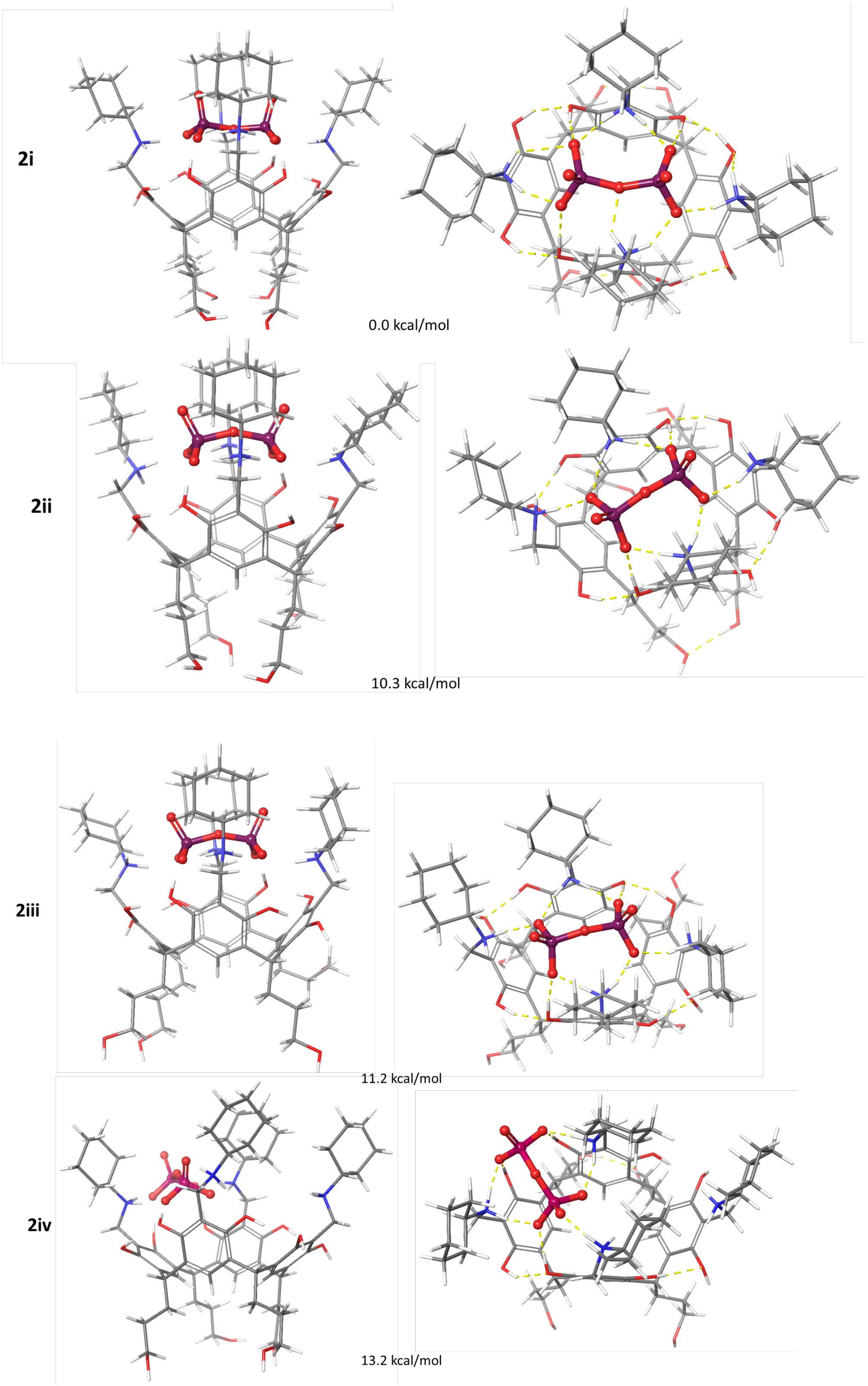

Figure S21. The lowest energy conformer of PPi@Cy-NAR (**2i-2iv**): side view and top view of same within 15 kcal of the global minimum.

11 References

1 A. Shivanyuk, T. P. Spaniol, K. Rissanen, E. Kolehmainen, V. Böhmer, *Angew. Chem., Int. Ed.*, 2000, **39**, 3497–3500.

2 N. K. Beyeh, M. Cetina, M. Löfman, M. Luostarinen, A. Shivanyuk, K. Rissanen, *Supramol. Chem.*, 2010, **22**, 737–750.

3 N. K. Beyeh, F. Pan, R. H. A. Ras, *Asian J. Org. Chem.*, 2016, **5**, 1027–1032.

4 N. K. Beyeh, H. H. Jo, I. Kolesnichenko, F. Pan, E. V. Anslyn, R. H. A. Ras, K. Rissanen, *J. Org. Chem.*, 2017, **82**, 5198–5203.

5 (a) P. Thordarson, *Chem. Soc. Rev.*, 2011, **40**, 1305–1323; (b) D. B. Hibbert and P. Thordarson, *Chem. Commun.*, 2016, **52**, 12792–12805.

6 N. K. Beyeh, F. Pan, S. Bhowmik, T. Mäkelä, R. H. A. Ras, K. Rissanen, *Chem. Eur. J.*, 2016, **22**, 1355–1361;

7 a) Schrödinger Release 2017-2: MacroModel, Schrödinger, LLC, New York, NY, 2017. b) Schrödinger Release 2017-2: Maestro, Schrödinger, LLC, New York, NY, 2017.

8 Z. Yu, M. P. Jacobson, J. Josovitz, C. S. Rapp, R. A. Friesner, *J. Phys. Chem. B* 2004, **108**, 6643-6654.

9 A. D. Bochevarov, E. Harder, T. F. Hughes, J. R. Greenwood, D. A. Braden, D. M. Philipp, D. Rinaldo, M. D. Halls, J. Zhang, R. A. Friesner, *Int. J. Quantum Chem.*, 2013, **113**, 2110–2142.

10 J. Contreras-García, E. R. Johnson, S. Keinan, R. Chaudret, J-P. Piquemal, D. N. Beratan, W. Yang, *J. Chem. Theory Comput.*, 2011, **7**, 625–632.
